# A systematic review of clinic and community intervention to increase fecal testing for colorectal cancer in rural and low-income populations in the United States – How, what and when?

**DOI:** 10.1186/s12885-017-3813-4

**Published:** 2018-01-06

**Authors:** Melinda M. Davis, Michele Freeman, Jackilen Shannon, Gloria D. Coronado, Kurt C. Stange, Jeanne-Marie Guise, Stephanie B. Wheeler, David I. Buckley

**Affiliations:** 1 Oregon Rural Practice-based Research Network (ORPRN), Oregon Health & Science University, 3181 SW Sam Jackson Park Rd, Mail Code: L222, Portland, 97239 OR USA; 2 Department of Family Medicine & OHSU-PSU School of Public Health, Oregon Health & Science University, Portland, OR USA; 3 VA Evidence-based Synthesis Program, Portland VA Medical Center, Portland, OR USA; 4 OHSU-PSU School of Public Health and Oregon Clinical and Translational Research Institute, Oregon Health & Science University, Portland, OR USA; 5 Center for Health Research, Kaiser Permanente, Portland, OR USA; 6 Center for Community Health Integration, Departments of Family Medicine & Community Health, Population & Quantitative Health Sciences, Sociology, and the Case Comprehensive Cancer Center, Case Western Reserve University, Cleveland, OH USA; 7 Departments of Obstetrics & Gynecology, Medical Informatics and Clinical Epidemiology, and Emergency Medicine and the OHSU-PSU School of Public Health, Oregon Health & Science University, Portland, OR USA; 8 Department of Health Policy & Management, Gillings School of Global Public Health, University of North Carolina, Chapel Hill, NC USA; 9 Departments of Family Medicine, Medical Informatics and Clinical Epidemiology, Public Health & Preventive Medicine, Oregon Health & Science University, Portland, OR USA

**Keywords:** Systematic review, Colorectal cancer, Fecal testing, Implementation science, Rural, Vulnerable populations

## Abstract

**Background:**

Interventions to improve fecal testing for colorectal cancer (CRC) exist, but are not yet routine practice. We conducted this systematic review to determine how implementation strategies and contextual factors influenced the uptake of interventions to increase Fecal Immunochemical Tests (FIT) and Fecal Occult Blood Testing (FOBT) for CRC in rural and low-income populations in the United States.

**Methods:**

We searched Medline and the Cochrane Library from January 1998 through July 2016, and Scopus and clinicaltrials.gov through March 2015, for original articles of interventions to increase fecal testing for CRC. Two reviewers independently screened abstracts, reviewed full-text articles, extracted data and performed quality assessments. A qualitative synthesis described the relationship between changes in fecal testing rates for CRC, intervention components, implementation strategies, and contextual factors. A technical expert panel of primary care professionals, health system leaders, and academicians guided this work.

**Results:**

Of 4218 citations initially identified, 27 unique studies reported in 29 publications met inclusion criteria. Studies were conducted in primary care (*n* = 20, 74.1%), community (*n* = 5, 18.5%), or both (n = 2, 7.4%) settings. All studies (*n* = 27, 100.0%) described multicomponent interventions. In clinic based studies, components that occurred most frequently among the highly effective/effective study arms were provision of kits by direct mail, use of a pre-addressed stamped envelope, client reminders, and provider ordered in-clinic distribution. Interventions were delivered by clinic staff/community members (*n* = 10, 37.0%), research staff (*n* = 6, 22.2%), both (n = 10, 37.0%), or it was unclear (n = 1, 3.7%). Over half of the studies lacked information on training or monitoring intervention fidelity (*n* = 15, 55.6%).

**Conclusions:**

Studies to improve FIT/FOBT in rural and low-income populations utilized multicomponent interventions. The provision of kits through the mail, use of pre-addressed stamped envelopes, client reminders and in-clinic distribution appeared most frequently in the highly effective/effective clinic-based study arms. Few studies described contextual factors or implementation strategies. More robust application of guidelines to support reporting on methods to select, adapt and implement interventions can help end users determine not just *which interventions work* to improve CRC screening, but *which interventions would work best in their setting given specific patient populations, clinical settings, and community characteristics*.

**Trial registration:**

In accordance with PRISMA guidelines, our systematic review protocol was registered with PROSPERO, the international prospective register of systematic reviews, on April 16, 2015 (registration number CRD42015019557).

**Electronic supplementary material:**

The online version of this article (10.1186/s12885-017-3813-4) contains supplementary material, which is available to authorized users.

## Background

Colorectal cancer (CRC) is the third most common cancer, and the second leading cause of cancer deaths, in the United States [1]. Unequivocal evidence demonstrates that guideline concordant screening decreases CRC incidence and mortality by 30–60% [2]. Numerous modalities are currently recommended for CRC screening in average risk adults aged 50–75 years, including colonoscopy every 10 years or a fecal occult blood test (FOBT) or fecal immunochemical test (FIT) within the past year [3–5]. However, current CRC screening rates are 63% across the United States [6], well below targets set by the National CRC Roundtable (80% by 2018) [7] and by Healthy People 2020 (70.5%) [8]. More striking are the consistent disparities in CRC screening in rural areas, among adults with low income, and in racial and ethnic minorities [6, 9–12]. Although improving, screening rates in these vulnerable populations may be 15%–30% lower than their non-rural, higher income, non-minority counterparts [13].

Multiple systematic reviews identify interventions that effectively increase CRC screening [14–16]. In 2016, the Community Preventive Services Task Force recommended the use of multicomponent interventions to increase screening for CRC. Multicomponent interventions combine two or more approaches to increase community demand (e.g., client reminders, small media), community access (e.g., reducing client costs), or provider delivery of screening services (e.g., provider reminders) or two or more approaches to reduce different structural barriers [17]. However, understanding how these interventions can be best implemented and in what populations (e.g., screening naïve versus experienced) and settings (e.g., rural versus urban settings, health system versus independent clinics) remains a neglected area of study [14, 18, 19].

Implementing interventions into routine care in clinic and community-based settings often involves the active engagement of multiple stakeholders and the adaptation of program elements to local contexts. In Oregon and elsewhere, primary care and health plan leaders are eager to identify, adapt, and implement interventions to improve CRC screening in order to achieve state performance benchmarks and to improve patient quality and experience of care. However, our work with primary care and health system partners found that stakeholders are interested not just in *which interventions work* to improve CRC screening, but *which interventions would work best in their setting given specific patient populations, clinical settings, and community characteristics* (see www.communityresearchalliance.org).

**Fig. 1:**
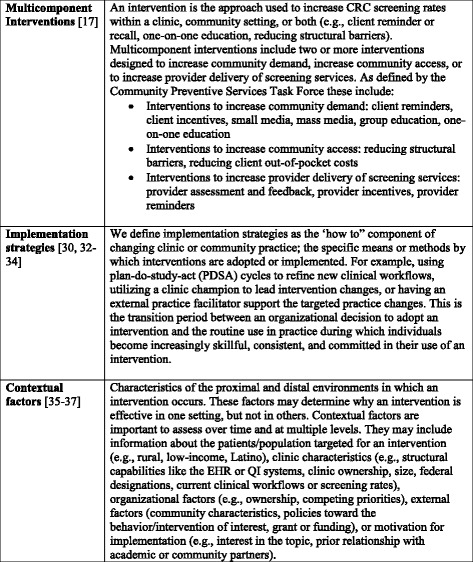
Definitions of Key Terms: Multicomponent Interventions, Implementation Strategies, and Contextual Factors

Therefore, we designed this systematic review to compare the effectiveness of interventions to improve fecal testing for CRC in clinic and community settings serving rural, low-income populations and their associated implementation strategies and contextual factors (see definitions and sources, Fig. 1) [17, 30, 32–37]. Determining how implementation strategies and contextual factors influence the uptake of interventions to increase fecal testing for CRC may help stakeholders identify the interventions best suited for use in their local settings.

We focused on fecal testing because this modality plays an important role in early detection of CRC, particularly in population groups at risk for experiencing disparities. Despite the increasing transition to FIT as the preferred modality for fecal testing due to superior adherence, usability, and accuracy [20, 21], we included studies of FOBT as well based on feedback from our technical expert panel (which included primary care professionals, health system leaders, and academicians) that early research on interventions to increase FOBT would likely inform current efforts to increase FIT [22–24]. Our key questions were:

1. What is the effectiveness of various interventions to increase CRC screening with FIT/FOBT compared with other interventions or usual care in rural or low-income populations?
2. How do implementation strategies (e.g., clinician champions, external practice facilitation) influence the effectiveness of interventions to increase FIT/FOBT screening for CRC in rural or low-income populations?
3. How do contextual factors (e.g., patient, clinic, community features) influence the effectiveness of interventions to increase FIT/FOBT screening for CRC in rural or low-income populations?
4. What are the adverse effects of interventions to increase FIT/FOBT screening for CRC in rural or low-income populations?

## Methods

We followed systematic review methods described in the Cochrane Handbook for Systematic Reviews of Interventions [25] and AHRQ [26]. The review was guided by a technical expert panel of primary care professionals, health system leaders, and academicians. Our protocol was registered with PROSPERO, the international prospective register of systematic reviews (registration number CRD42015019557). The review is reported in accordance with the PRISMA publication standards [27–29].

### Search strategy

We developed our search strategy with a research librarian with keywords for colorectal cancer, screening, stool, and FIT or FOBT (Additional file 1: Appendix A). We searched MEDLINE®, Cochrane Library, Scopus, and clinicaltrials.gov from January 1, 1998 through March 31, 2015, and updated our search of MEDLINE® and the Cochrane Library on July 19, 2016. Additionally, we reviewed reference lists of included studies and relevant systematic reviews.

### Study selection

We screened studies using specific inclusion criteria detailed in Additional file 1: Appendix B. Included studies targeted patients aged 50–75 years, occurred in settings serving rural, Medicaid, or lower socioeconomic status populations in the United States, and reported outcomes for FOBT/FIT screening for CRC. We included randomized controlled trials, non-randomized controlled trials, cohort studies, and pre-post studies. Two research members (MMD, MF) screened titles and abstracts for eligibility; and then obtained the full-text of potentially eligible citations for further evaluation. Two investigators independently reviewed the full articles to determine final inclusion, with differences resolved through consensus or inclusion of a third investigator.

### Data abstraction

Data from included studies were abstracted into a customized Microsoft Excel Spreadsheet by one investigator and reviewed for accuracy and completeness by a second investigator. Our overall analytic framework was informed by the Consolidated Framework for Implementation Research (CFIR) [30] and recent reviews on complex multicomponent interventions [18, 31]. Information was abstracted from each study on study setting, design, intervention attributes (e.g., intervention arms tested, type of FIT/FOBT used, theoretical framework), and study results (CRC screening rates, impact by intervention component, recruitment success). Additionally, for each intervention arm tested in an included study we categorized intervention components into distinct, individual categories starting with the Community Preventive Services Task Force recommendations for CRC screening (see Fig. 1) and refined based on study findings (e.g., 5 individual sub-categories identified under reducing structural barriers) [17].

We attempted to abstract data related to implementation strategies and contextual factors when feasible (see Fig. 1). Specifically, we tried to catalogue implementation strategies according to recent work by Proctor, Powell, and colleagues, which encourages the identification of discrete strategies and documentation of elements such as the actor, action, action targets, dose, and theoretical justification [30, 32–34]. We also attempted to classify contextual factors based on work by Stange and colleagues focused on the identification of factors across multiple levels (e.g., patient, practice, organization, and environment), the motivation for the intervention, and change in context over time [35–37]. However, a paucity of detail in the manuscripts led us to abstract any information regarding implementation strategies and contextual factors in a figure summary, rather than as discrete components as originally planned. Disagreements were resolved by discussion; one author (DIB) adjudicated decisions as needed.

### Risk of bias/quality assessment

Two authors independently assessed the quality of each included study. We used a tool developed by the Cochrane Collaboration for randomized controlled trials and control trial designs [25]. We used a tool developed by the National Institutes of Health for quality assessment in pre-post studies which included questions about pre-specification of study details (e.g., aims, eligibility criteria, outcome measures) and methods for data collection and analysis (e.g., method for outcome assessment, analysis controlled for clustering) [38, 39] We did not assess the quality of feasibility studies because no validated criteria are available. Rated studies were given an overall summary assessment of “low”, “high”, or “unclear” risk of bias. Disagreements were resolved through discussion.

### Data synthesis

We constructed evidence tables showing the study characteristics and results for included studies by key question. We clustered studies based on the (1) intervention setting: primary care clinic, community-based settings, or both and (2) intervention components used in each study arm (e.g., client reminder or recall, small media, provider incentives). We assessed the effectiveness of the study arms based on the percent of CRC screening completion in the intervention arms compared with the control/usual care arm. We categorized the study arms as being highly effective if screening at the end of follow-up was higher in treatment than the usual care/control condition by more than 25%; effective if the difference was 10–25%; marginally effective if there was an increase of less than 10%; or having no effect. We developed this categorization based on the proportional distribution of the outcome and author assessments of clinical significance. We then assessed which intervention components occurred more frequently among the effective and highly effective study arms, compared with intervention arms that had little or no effect. Finally, we assessed studies to compare their intervention components, implementation strategies, contextual factors, methods, and findings. We compiled a summary of findings for each key question and drew conclusions based on qualitative synthesis of the findings. We did not combine the studies in a quantitative manner via meta-analysis because of the heterogeneity of interventions, methods, and settings.

### Rating the body of evidence

We attempted to grade the overall strength of the evidence as high, moderate, low, or insufficient using a method developed by AHRQ [26]. This method considers the consistency, coherence, and applicability of a body of evidence, as well as the internal validity of individual studies. However, because the studies used multicomponent interventions and did not assess the effectiveness of individual components, we were limited from applying AHRQ criteria to the strength of evidence for the individual intervention components.

## Results

**Fig. 2:**
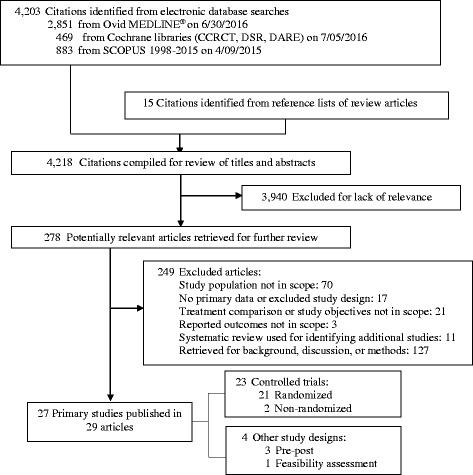
Literature Flow Diagram

The combined literature searches initially yielded 4218 titles and abstracts, including 4203 from electronic database searches, and 15 from reference lists of systematic reviews and other relevant articles. As summarized in Fig. 2, we assessed 278 full-text articles for eligibility, of which 27 studies reported in 29 publications met inclusion criteria. We identified 20 RCTs (published in 21 articles [40–60], 2 non-randomized controlled trials [61, 62], 3 pre-post studies [63–65], 1 cohort study [66] and 1 feasibility study [67] that contained primary data relevant to the key questions. The descriptive characteristics and findings of the 27 included studies are found in Additional file 1: Appendix D, Table D1.

Studies occurred in primary care clinics (*n* = 20, 74.1%), communities (*n* = 5, 18.5%), or in both settings (n = 2, 7.4%). Over half of the studies used FOBT (*n* = 18, 66.6%); FIT was used in 8 studies (29.6%); one study used both FIT and FOBT (3.7%). As summarized in Additional file 1: Appendix D, Table D2, patient eligibility and the informed consent process varied across the studies. Of the 20 clinic-based studies, 5 (25%) did not report on the patient consent process, 8 (40%) received a waiver of informed consent, 4 (20%) utilized an informed consent process, 2 (10%) used opt-out, and 1 was conducted as a quality improvement study (5%). Six of the seven studies (85.7%) conducted in community or both clinic/community settings did not report on the patient consent process; the one remaining study required informed consent. Many of the included studies rated as high or unclear risk of bias, this was often related to insufficient detail in the methods as well as lack of blinding. Quality assessment details appear in Additional file 1: Appendix C, Tables C1 and C2.

### Interventions and effectiveness

#### Study-level

All studies (*n* = 27, 100.0%) described multicomponent interventions. As presented in Additional file 1: Appendix D, Table D3, the majority of studies used some form of strategy to increase community demand (*n* = 25, 92.6%) and/or to increase community access (*n* = 24, 88.9%) in the most complex intervention arm. Commonly used intervention components to increase community demand included small media (*n* = 17, 63.0%), client reminder or recall (*n* = 16, 59.3%), and one-on-one education (*n* = 14, 51.9%). Strategies to reduce structural barriers included increasing in-clinic distribution of FIT/FOBT by providers (*n* = 11, 40.7%) or clinical staff/research team members (*n* = 12, 44.4%), programs that mailed FIT/FOBT materials directly to the patient’s home (*n* = 10, 37.0%, aka “direct mail programs”), provision of pre-addressed stamped envelopes to facilitate return of the completed FIT/FOBT (*n* = 12, 44.4.7%), or FIT/FOBT distribution by participant request (*n* = 4, 14.8%). Each of the 10 studies that used a direct mail program also utilized client reminder and recall. Although 12 studies used pre-addressed stamped envelopes, this intervention component was only employed in 70% (7/10) of the studies using a direct mail program (see Additional file 1: Appendix D, Table D3). Many studies also used patient navigators (n = 12, 44.4%) or other miscellaneous intervention components (*n* = 17, 63.0%) such as by providing culturally tailored materials or having participants complete surveys about CRC screening knowledge and behaviors.

#### Study arm-level

As summarized in Table 1, most studies tested multicomponent interventions in varied combinations across multiple study arms. Among the 20 clinic-based studies [40, 43–58, 60, 61, 64, 66], there were 27 active treatment arms. The most frequently used intervention components in clinic-based treatment arms included client reminder or recall (16/27, 59.3%,), small media (16/27, 59.3%), provision of pre-addressed stamped envelopes (15/27, 55.6%), and direct mail programs (13/27, 48.1%). No clinic-based treatment arms used client incentives, mass media, or group education. Over half (60%, 12/20) of the control arms in the clinic-based studies distributed FIT/FOBT kits upon order by clinicians as part of usual care.

**Table 1:** Number of study arms using specified intervention components among clinic- based, community-based, and both clinic/community-based studies

Intervention components grouped by strategic aim	Active intervention arms grouped by study setting			Referent group (usual care/ control arm)
	Clinic-based	Community-based	Combined Clinic/ Community-based	
Increase Community Demand				
Client reminder or recall	16	1	2	1
Client incentives	0	0	1	0
Small media	16	5	4	3
Mass media	0	0	2	0
Group education	0	6	1	1
One-on-one education	11	4	3	3
Increase Community Access				
Reducing structural barriers				
▪ Provider ordered in-clinic distribution	14	1	0	12
▪ Systematic distribution by clinic staff or study team	10	2	3	7
▪ Direct mail	13	0	0	0
▪ Pre-addressed stamped envelope provided	15	1	0	1
▪ Kit available by participant request	2	1	1	1
Reducing client out-of-pocket costs	1	2	4	1
Increase Provider Delivery of Screening Services				
Provider assessment and feedback	3	0	0	2
Provider incentives	2	0	0	2
Provider reminder and recall systems	6	0	0	1
Other				
Patient navigators	10	1	4	0
Patient questionnaires or surveys about CRC screening knowledge and behaviors	5	2	0	6
Materials tailored for specific cultures or low literacy	11	0	0	2
Leveraging social networks	0	4	0	0
Total number of studies	20	5	2	*
Total number of study arms	27	7	4	*

* N control arms: *N* = 20 among 20-clinic based studies; *N* = 4 among 5 community-based studies; *N* = 0 in 2 combined clinic/community-based studies

Among the seven studies conducted either in community settings [41, 42, 59, 63, 65] or that targeted both clinic and community settings [62, 67], there were 11 active treatment arms. Group education (*n* = 7, 63.6%), small media (*n* = 9, 81.8%), and one-on-one education (n = 7, 63.6%) were the most frequently used intervention components used in these settings. The strategy of leveraging social networks was only used in community-based studies.

All studies that included highly effective treatment arms were clinic-based. In these clinic-based studies, 9 treatment arms were rated as highly effective (greater than 25% higher screening rate compared with the control/usual care group), and 12 treatment arms were rated as effective (10% to 25% higher screening rate compared with control/usual care group). As detailed in Table 2, the components that occurred most frequently among the highly effective and effective study arms were provision of kits by direct mail, provision of a pre-addressed stamped envelope, client reminder or recall, and provider ordered in-clinic distribution. Additionally, some studies used a layered approach, by adding intervention components among multiple study arms, or by adding successive interventions within a single intervention arm in an attempt to reach non-responders. Studies with highly effective treatment arms occurred in a variety of ethnic populations, however, most of these studies occurred in urban areas (6 of 7 studies).

**Table 2:** Effectiveness of intervention components to improve fecal testing for CRC among clinic-based study arms, *N* (%)

Intervention components grouped by strategic aim	Active intervention arms grouped by effectiveness, N (%)^a^			Referent group (usual care/ control arms)
	Highly effective	Effective	Marginal/null effect	
Increase Community Demand^**b**^				
Client reminder or recall	7 (77.8)	7 (58.3)	2 (33.3)	0 (0.0)
Small media	5 (55.6)	6 (50.0)	5 (83.3)	2 (10.0)
One-on-one education	3 (33.3)	4 (33.3)	4 (66.7)	2 (10.0)
Increase Community Access				
Reducing structural barriers				
▪ Provider ordered in-clinic distribution	7 (77.8)	6 (50.0)	1 (16.7)	12 (60.0)
▪ Systematic distribution by clinic staff study team	2 (22.2)	5 (41.7)	3 (50.0)	6 (30.0)
▪ Direct mail	8 (88.9)	5 (41.7)	0 (0.0)	0 (0.0)
▪ Pre-addressed stamped envelope provided	8 (88.9)	5 (41.7)	2 (33.3)	1 (5.0)
▪ Kit available by participant request	1 (11.1)	0 (0.0)	1 (16.7)	1 (5.0)
Reducing out-of-pocket costs	0 (0.0)	1 (8.3)	0 (0.0)	1 (5.0)
Increase Provider Delivery of Screening Services				
Provider assessment and feedback	1 (11.1)	2 (16.7)	0 (0.0)	2 (10.0)
Provider incentives	1 (11.1)	1 (8.3)	0 (0.0)	2 (10.0)
Provider reminder and recall systems	3 (33.3)	2 (16.7)	1 (16.7)	1 (5.0)
Other ^c^				
Patient navigators	4 (44.4)	4 (33.3)	2 (33.3)	0 (0.0)
Patient questionnaires or surveys about CRC screening knowledge and behaviors	0 (0.0)	2 (16.7)	3 (50.0)	3 (15.0)
Materials tailored for specific cultures or low literacy	5 (55.6)	5 (41.7)	1 (16.7)	2 (10.0)
Total combined study arms in 20 clinic-based studies	9 (100.0)	12 (100.0)	6 (100.0)	20 (100.0)
Average number of intervention components per study arm	6.0	4.6	4.0	1.7

^a^ Percent of the total number of study arms in each column

^b^ We do not report on client incentives, mass media, group education because these intervention components were not used in clinic-based studies

^c^ We do not report on leveraging social networks because this intervention component was not used in clinic-based studies

Among 10 studies that used direct mailing of the FIT/FOBT as an intervention strategy [40, 43, 46, 47, 49, 50, 52, 54, 55, 58, 61], the mailed kit plus 1–3 phone reminders were more effective than usual care in increasing FIT/FOBT use; the frequency of FIT/FOBT completion in the intervention arms ranged from 29% to 82% compared with 1.1% to 37% in the control arms. Intensive outreach using navigators (e.g., home visits, phone calls) was used as a core component of some study interventions [49, 51–53], as a more complex study intervention arm [44, 54, 55, 61], or as a technique for reaching non-responders in layered interventions [40, 47]. FIT/FOBT completion among non-responders generally remained lower after outreach compared with early responders. The overall effectiveness of intensive outreach compared with minimal or automated phone/text outreach was not consistent across studies. Our data suggest that effectiveness may relate to the timing at which navigation was delivered (e.g., early on as a core component of the initial intervention or as a follow-up strategy used with non-responders to another intervention).

### Contextual factors

Few studies provided details on contextual factors beyond the study location or the population of interest, see summary in Table 3 (details in Additional file 1: Appendix D, Table D4). Twenty (74%) of the 27 studies were conducted in urban settings, five studies (18.5%) were conducted in rural settings and one did not designate a geographic setting (3.7%). Of the five studies conducted in rural settings, three occurred in primary care clinics and two were based in the community. Of the 20 studies conducted in clinics, 14 (70.0%) occurred in clinics affiliated with a larger system (e.g., hospital, FQHC, health department), two occurred in clinics affiliated with practice-based research networks (10.0%), two in independent clinics (10.0%), and two could not be determined (10.0%). Irrespective of study setting as detailed in Additional file 1: Appendix D, Table D4, very limited details, if any, were provided about organizational priorities or environmental factors that may have influenced a site’s interest in participating in the study. A few studies built on existing community-based programs [65], quality improvement initiatives [40, 47], or emerged based on community or health system identified needs [62, 67].

**Table 3:** Summary of Contextual Factors and Implementation Strategies

Study	Participant characteristics			N clinics or sites		Study site characteristics			Implementation strategies		
	Race/ Ethnicity^a^	Screen-ing status	Socio-economic indicators	Health systems^b^	PBRN affiliated/ Other	EHR System	Baseline CRC screening	Rural or urban	Development	Training and/or monitoring	Delivered by
Clinic-based Studies											
Baker, 2014 [40]	Latinos	Prior FOBT	–	4	–	Yes, NOS	17% in 2007; 43% in 2009	Urban	To address known barriers	NR	Clinic
Coronado, 2011 [43]	Latinos	–	–	1	–	Yes, NOS	NR	Urban	Collaboratively designed	Training	Clinic
Coronado, 2014 [61]	–	Not up-to-date	Uninsured	1	–	Yes, NOS	5.1% in 2012	Urban	Clinic adapted workflows	Training, meetings	Both
Davis, 2013 [44]	–	Not up-to-date	Low-income	8	–	No, paper charts	1–2% at Baseline	Rural	Focus groups & interviews	Training	Both
Friedman, 2001 [45]	African American	Not up-to-date	Low-income	–	1	Unclear	NR	Urban	NR	NR	External
Goldberg, 2004 [46]	African American	–	Low-income	1	–	Yes, NOS	NR	Urban	NR	NR	Both
Goldman, 2015 [47]	Latinos	Not up-to-date	–	8	–	Yes, GE Centricity	17% in 2007; 43% in 2009	Urban	Modeled after prior intervention	NR	Clinic
Greiner, 2014 [48]	–	Not up-to-date	Low-income	–	9	Unclear	NR	Urban	Interviews, usability testing	NR	External
Gupta, 2013 [49]	–	Not up-to-date	Uninsured	13	–	Unclear	NR	Urban	NR	NR	Unclear
Hendren, 2014 [50]	Ethnic minority	–	Low-income	–	1	Yes, NOS	NR	Urban	NR	NR	Both
Jandorf, 2005 [51]	–	Not up-to-date	Low-income	1	–	Unclear	NR	Urban	Focus groups	NR	External
Jean-Jacques, 2012 [52]	Ethnic minority	Not up-to-date	Low-income; uninsured	1	–	Yes, NOS	17% in 2008; 36% in 2009	Urban	NR	Training, supervision	Both
Lasser, 2011 [53]	Multi-cultural	Not up-to-date	Low-income	–	6, PBRN	Yes, EPIC	NR	Urban	Prior research, piloting	Training, meetings	External
Levy, 2012 [54]; Levy, 2013 [55]	–	Not up-to-date	–	–	16, PBRN	Both paper and EHR, NOS	54.3% at baseline	Rural	NR	NR	External
Potter, 2011 [56]	Multi-cultural	Not up-to-date	–	6	–	Both paper and EHR, NOS	NR	Urban	Piloted intervention	Training, observation visits	Clinic
Potter, 2011 [66]	Chinese-American	Not up-to-date	Low-income	1	–	Yes, NOS	NR	Urban	Tailored with clinic leader input	Training, observation visits	Clinic
Roetzheim, 2004 [57]	–	–	Uninsured	–	8	Unclear	NR	Rural	NR	Training, audits & feedback	Clinic
Singal, 2016 [58]	Multi-cultural	Not up-to-date	Low-income	12	–	Unclear	NR	Urban	NR	Training	External
Tu, 2006 [60]	Chinese	–	–	1	–	Unclear	NR	Urban	Interviews, focus groups	NR	Both
Tu, 2014 [64]	Vietnamese & Chinese	–	–	2	–	Yes, ICHS EMR	NR	Urban	Adapted from prior intervention	Training	Both
Community-based Studies											
Braun, 2005 [41]	Native Hawaii-ans	–	–	–	16 civic clubs	No	NR	NR	Surveys, focus groups	NR	Both
Campbell, 2004 [42]	African American	–	–	–	12 churches	No	NR	Rural	Focus groups	Training	Com-munity
Larkey, 2006 [63]	Latino	–	–	–	Churches & com-munity orgs (*N* = NR)	No	NR	Urban	Staff developed	NR	Com-munity
Thompson, 2006 [59]	Latino	–	–	–	20 agricultural communities	No	NR	Rural	Focus groups, community advisory board	Training	Com-munity
Wu, 2010 [65]	Asian Americans	–	–	–	NR	No	NR	Urban	Expanded existing program	NR	Both
Combined Clinic- and Community-based Studies											
Redwood, 2011 [67]	NR	–	Low-income; uninsured	–	1 County	No	NR	Urban	Refined over time	NR	Com-munity
Sarfaty, 2005 [62]; Sarfaty, 2006 [68]	Ethnically diverse	–	Low-income; uninsured	–	1 County	No	NR	Urban	By state	NR	Both

*NOS* not otherwise specified, *NR* Not reported, *PBRN* Practice-based Research Network

^a^ Predominant minority race/ethnicity of study sample

^b^ May include clinics that are associated with the following systems: Hospital, Federally Qualified Health Center (FQHC), health department

Thirteen out of the 27 studies (48.1%) targeted patients of a specific race or ethnic group [40–43, 45–47, 59, 60, 62, 63, 65, 66] and six (22.2%) occurred in clinics that largely served racial/ethnic minority or multicultural patients [50, 52, 53, 56, 58, 64]. Five studies focused on Latino populations, four were conducted in Asian patient populations, three focused on African Americans, and one study was conducted among native Hawaiians [41]. Of the six studies conducted in community settings or both clinic/community settings, none reported on the prior screening history of eligible patients (0%). Of the 20 clinic-based studies, 13 (65.0%) targeted patients who were not-up-to-date with CRC screening [44, 45, 47–49, 51–56, 58, 61, 66]. Six studies (30.0%) did not specify participant’s prior screening history and one (5.0%) targeted patients who had completed a prior FOBT. Six of the 20 clinic-based studies (30.0%) reported on CRC screening rates within a clinic or health system prior to the intervention [40, 44, 47, 52, 54, 55, 61], and two of these studies occurred in the same health system [40, 47]. Baseline screening rates in these settings varied widely, from 1 to 2% to 54.3%. In one study setting, the health system had initiated work to improve CRC screening prior to the current studies by reducing structural barriers, introducing audit and feedback, implementing provider reminders, and using CRC as a quality metric by which providers received incentive payments. These changes had increased CRC screening from 17% in 2007 to 43% in 2009 prior to any interventions facilitated by the study teams [40, 47].

None of the studies in community or both community/clinic settings reported on use of an electronic health record (EHR) system. In the 20 clinic-based studies, 10 had an EHR (3 specified, 7 unspecified), 7 were unclear, 2 used both paper and an EHR, and 1 used paper charts (see Table 3 and details in Additional file 1: Appendix D, Table D4).

### Implementation strategies

As summarized in Table 3 and detailed in Additional file 1: Appendix D, Table D5, studies provided very limited information on the methods used to encourage the adoption of the interventions into practice; it was often hard to determine if any implementation support occurred. Among clinic-based interventions, 8 (40.0%) did not report how the intervention was developed, 4 (20.0%) used focus groups, interviews, or surveys to inform intervention development, 4 (20.0%) were based on a pilot or prior intervention, and 4 (20.0%) were informed by stakeholder input or other methods. In community and community/clinic-based studies, intervention development was informed either by focus groups, interviews, or surveys (*n* = 3, 42.9%) or by stakeholder input or other methods (*n* = 4, 57.1%).

Methods to support training on the intervention and monitoring fidelity of the intervention components over time was not reported for over half of the 27 studies (*n* = 15, 55.6%). Although training was mentioned in 12 of these studies, the details provided were often limited and varied widely: 6 (50.0%) did not report on the length of training, 4 (33.3%) had one session lasting 30 min to 2 h, 1 (8.3%) had a 2 day training, and 1 (8.3%) delivered multiple in-service presentations to the medical assistants or clinic staff. Potter et al. provided the most robust description of their training which included a 1-h training for nursing staff; onsite review of study procedures with individual nurses; frequent site-visits to ensure nursing staff were aware of the week’s study protocol; and daily availability of research assistants to ask questions regarding study implementation [56]. Only six studies (22.2%) provided any data on monitoring intervention fidelity, which could include meetings, observation visits, supervision, or random audits with compliance feedback [52, 53, 56, 57, 61, 66].

Interventions were delivered by clinic staff, community members, the research team, or a combination. Delivery was unclear in one clinic-based study, the others were almost equally distributed across the three categories: 7 (35.0%) were delivered by both clinic staff and the research team, 6 (30.0%) by clinic staff, and 6 (30.0%) by the research team. Community or community/clinic studies were delivered by community members (*n* = 4, 57.1%) or by both community members and the research team (*n* = 3, 42.9%). While few studies discussed strategies to support implementation over time, Roetzheim et al. [57] described routine feedback sessions with research members and clinical staff 6 and 12 months after the intervention had been implemented to discuss intervention progress, challenges occurring, and what could be done to improve implementation.

Seven studies provided highly heterogeneous information on intervention costs [40, 42, 44, 46, 50, 57, 68], see Additional file 1: Appendix D, Table D5. Some studies providing data on the cost of individual intervention components and others calculating the cost per patient or per patients screened. Three studies determined intervention costs per patient screened, which ranged from $43 to $1688 [40, 44, 68].

## Discussion

We found that studies to promote fecal testing in rural and low-income populations used multicomponent interventions that were tested in varied combinations across multiple study arms. Over half of the studies (16/27, 59.3%) used an informed consent process or did not provide details on the consent process, leaving it difficult to interpret if the participating patients were “different” than the general population served by the clinic or in the community. The most frequently used interventions in clinics included client reminder or recall, small media, provision of pre-addressed stamped envelopes for FIT/FOBT return, and direct mail programs. Many studies also employed outreach using navigators. In clinic-based studies, the components that occurred most frequently among the highly effective and effective study arms were provision of kits by direct mail, use of a pre-addressed stamped envelope, client reminders, and provider ordered in-clinic distribution.

Few studies included an adequate description of the contextual factors and approaches used to implement the intervention needed for health system leaders, primary care providers, or researchers to replicate the study in their own settings or to determine if the intervention was a good “fit” for the local context. For example, only 25.9% (7/27) reported on baseline screening rates prior to study implementation and only three clinic-based studies (15.0%, 3/19) named the EHR system used. In relation to implementation factors, delivery of the interventions occurred by clinic/community staff, research team members, or both – yet over half of the studies did not report on the type or length of training used. Few studies indicated if new clinical staff were hired or additional resources were provided to support intervention implementation. Seven studies reported on intervention costs, yet methods were heterogeneous, estimates highly variable, and details inadequate for determining how much an intervention might cost in a target setting. Only six studies described monitoring intervention fidelity, which was often limited to an acknowledgement that some form of interaction between the study team and clinical staff occurred (e.g., meetings, supervision).

Our review describes the effectiveness of interventions to improve fecal testing in low-income and rural patients in clinic and community-based settings. As in the recent review on CRC interventions by the Community Preventive Services Task Force, we found that most interventions tested in these settings were multicomponent in nature (i.e., included two or more strategies) [17]. Strategies to increase community demand (i.e., client reminders) as well as to increase community access (i.e., direct mail, use of a pre-addressed stamped envelope, in-clinic distribution) were intervention components commonly found in highly effective/effective study arms tested in clinic settings. However, generating precise estimates of specific intervention components was difficult in part because of the limited information on baseline screening levels for participating clinics, different patient targets (e.g., prior FIT/FOBT versus screening naïve), and variation in the consent process (e.g., waived versus informed). Moreover, none of the articles included in our review explicitly compared implementation strategies or the impact of contextual factors on intervention effectiveness.

One key challenge identified by our review was that the articles frequently lacked information in relation to local contextual factors and implementation strategies that could be used to inform end users as to which interventions would work best in their setting given specific patient populations, clinical settings, and community characteristics – and how to implement them. In their recent review to develop a taxonomy for CRC screening promotion, Ritvo and colleagues emphasized the need to describe the engagement sponsor, population targeted, alternative screening tests, delivery methods, and support for test performance (EPADS) in future reporting [69]. Numerous recent articles suggest strategies and approaches that can improve reporting of intervention studies, including TIDieR (Template for Intervention Description and Replication [70]) or PARIHS (Promoting Action on Research Implementation in Health Services [71]). Moreover, recent work by Powell and colleagues identified 73 implementation strategies grouped in 9 categories; thus providing a common terminology that can be used to design and evaluate effectiveness as well as implementation research studies [32, 72]. However, as indicated in our review, this data is either not being gathered by research investigators or it is not being reported in publications.

We speculate that lack of reporting on the impact of implementation strategies and contextual factors may reflect funder preferences for novel discoveries, researcher bias toward intervention effectiveness measured by statistical significance rather than nuanced understanding of how, when, and why a specific intervention works, and journal restrictions on manuscript length. Our experience also suggests that pragmatic studies conducted in settings serving rural and vulnerable populations are often better equipped for implementing interventions owing to greater capacity for data reporting, workflow refinements, and leadership interest/engagement than would be needed to scale these interventions across average or “below average” settings. This presents challenges and opportunities for cancer control researchers and implementation scientists. Namely, it presents opportunities to compare the effectiveness of implementation strategies needed to translate effective multicomponent interventions into diverse clinic, community, and population settings; to use SMART designs to test stepped-implementation support in response to baseline setting capacity and intervention responsiveness over time; and to implement “layered” interventions which may first build clinic capacity and visit-based workflows prior to implementing population outreach programs [73].

We are seeing a shift to address these needs through funding opportunities through the Cancer Moonshot, in NCI and CDC’s continued support of collaborative research networks and implementation research, as well as the emergence of local initiatives in response to advanced payment models that reward improved CRC screening and disparities reduction [74, 75]. Taking the next step forward in implementation of evidence-based approaches to improve cancer screening also argues for investing in academic-community collaborations before research studies begin through the infrastructure of practice-based research networks [76], researcher in residence models [77], or participatory research methods [78, 79]. Such partnered approaches can help actualize the call for rapid and relevant science [80]. Shifting the research paradigm to support collaborative, partnered implementation enables leveraging “teachable moments” and “tipping points” when research evidence can be used to inform local, regional, or system-wide interventions that are under consideration or actively underway.

There are a few important limitations in the present study. First, we limited our review to studies targeting rural and low-income patients in the United States. These populations experience CRC screening disparities, and our stakeholder partners wanted to know what interventions worked best for settings serving these patients. It is also possible that interventions that are effective for these hard-to-reach populations need to be different or more intensive than those implemented in higher resourced populations. Second, we acknowledge that the requirement to have quantitative outcome data on changes in FIT/FOBT may have led to the exclusion of studies targeting any type of CRC screening or to studies focused on qualitative methods. Future systematic reviews on this topic would benefit from interviews with the lead or senior author or a realist review [81–83] which could generate a more contextualized understanding of how and why complex interventions achieve particular effects in particular contexts. Finally, we created cut-offs for evaluating the individual intervention components based on a proportional distribution of the outcomes. This approach identified four intervention components that commonly appeared in the highly effective/effective study arms in clinic based studies (i.e., direct mail, client reminders, addressed stamped envelopes, in-clinic distribution); the use of alternative cut-points may have identified additional strategies. Despite these limitations, our findings document the general deficiency in reporting on contextual factors, implementation strategies, and how these factors interact with the intervention. Our review provides a critical starting point for future reviews and informs research funding and publication in this area.

There is growing interest in the conduct and reporting of pragmatic research that can be used by stakeholders to inform practice in primary care and community settings. Such evidence can guide health systems administrators in selecting an appropriate intervention in their setting, as well as approaches for adapting a given intervention or implementation approach based on contextual factors. Interventions to improve FIT/FOBT for CRC screening should be considered generally as complex interventions (e.g., multicomponent, context-sensitive, and highly dependent on the behaviors of participants and providers) [84]. As the field of pragmatic research continues to advance, our findings suggest that the use of standard guidelines for the reporting of implementation strategies and contextual factors within trials may be useful. Building on the policy of the National Institute of Health (NIH) to require detailed on rigor and reproducibility in grant submissions [85], journals, editors, and funders could mandate scientists to more freely share protocols, implementation toolkits, or intervention websites. Tools used in our review may inform such guidelines, including more routine application of Proctor, Powell and colleague’s recommendations for tracking and reporting implementation strategies [32, 34, 72] or Stange and colleague’s conceptualization of contextual factors [35–37]. Rather than reducing the nuances of implementation in diverse, complex adaptive systems to soundbites, we need to apply the principles of precision medicine to research design to determine what works for whom and why, rather than what works after variation has been removed. Funders and journal editors should apply these policies so that investigation of how intervention effects are modified by context and implementation strategies moves from being a “new methodological frontier” to a standard of practice for research [86].

## Conclusion

Multicomponent interventions can effectively increase fecal testing for CRC across diverse rural and low-income communities. Few community based studies were effective, but this may be because they targeted patients irrespective of CRC screening eligibility. For clinic-based studies, the intervention components frequently found in highly effective and effective treatment arms included mailing FIT/FOBT materials directly to patient’s homes (i.e., “direct mail programs”), provision of a pre-addressed stamped envelope to facilitate kit return, client reminder and recall, and provider ordered in-clinic distribution. Based on the community guide, these strategies are designed to improve community access by reducing structural barriers and to increase community demand. Few studies described contextual factors or implementation strategies in the detail needed for stakeholders to clearly determine how and which interventions would work in their local contexts. More robust application of guidelines to support reporting of implementation strategies and contextual factors in funding and publication are needed so that stakeholders can determine not just *which interventions work* to improve CRC screening, but *which interventions would work best in their setting given specific patient populations, clinical settings, and community characteristics*.

## Additional file

Additional file 1: **Appendix A.** Search Strategies. **Appendix B.** Study Selection: Table of Inclusion/Exclusion Codes, Definitions and Key Questions. **Appendix C.** Quality Assessment: Quality Assessment for Randomized Controlled Trials (RTC) or Control Trial (CT) Designs, Quality Assessment for Studies using a Pre-Post Design. **Appendix D.** Data Supplement: **D1.** Characteristics and findings of included studies, stratified by intervention, **D2.** Participant recruitment and recruitment success in intervention studies to improve FIT/FOBT screening for CRC, stratified by intervention setting, **D3.** Intervention Components – Tracked for the most complex intervention arm tested in a study, **D4.** Contextual Factors in Interventions to Improve FIT/FOBT screening for CRC, stratified by setting, and **D5.** Implementation strategies used in interventions to improve FIT/FOBT CRC screening, stratified by setting. (DOCX 442 kb)

## Abbreviations

CFIR: Consolidated Framework for Implementation Research
CRC: Colorectal cancer
EHR: Electronic Health Record
EPADS: Engagement sponsor, population targeted, alternative screening tests, delivery methods, and support for test performance
FIT: Fecal Immunochemical Test
FOBT: Fecal Occult Blood Test
TIDieR: Template for Intervention Description and Replication
