# Supplementary material for: A systematic review of clinic and community intervention to increase fecal testing for colorectal cancer in rural and low-income populations in the United States – How, what and when?
Source: BMC Cancer. 2018 Jan 6;18:40. doi: 10.1186/s12885-017-3813-4 (PMC5756384; doi:10.1186/s12885-017-3813-4)
Supplement: Additional file 1: — Appendix A. Search Strategies. Appendix B. Study Selection: Table of Inclusion/Exclusion Codes, Definitions and Key Questions. Appendix C. Quality Assessment: Quality Assessment for Randomized Controlled Trials (RTC) or Control Trial (CT) Designs, Quality Assessment for Studies using a Pre-Post Design. Appendix D. Data Supplement: D1. Characteristics and findings of included studies, stratified by intervention, D2. Participant recruitment and recruitment success in intervention studies to improve FIT/FOBT screening for CRC, stratified by intervention setting, D3. Intervention Components – Tracked for the most complex intervention arm tested in a study, D4. Contextual Factors in Interventions to Improve FIT/FOBT screening for CRC, stratified by setting, and D5. Implementation strategies used in interventions to improve FIT/FOBT CRC screening, stratified by setting. (DOCX 442 kb) [file 12885_2017_3813_MOESM1_ESM.docx]

ONLINE SUPPLEMENT

**A Systematic Review of Clinic and Community Intervention to Increase Fecal Testing for Colorectal Cancer in Rural and Low-Income Populations in the United States –**

**How, What and When?**

Table of Contents

Appendix A. Search Strategies 2

Appendix B. Study Selection 4

Appendix C. Quality Assessment 7

Appendix D. Data Supplement

Table 1. Characteristics of included studies, stratified by intervention setting 9

Table 2. Participant recruitment and recruitment success in intervention studies, stratified by intervention setting 18

Table 3. Intervention Components – Tracked for the most complex version of an intervention23

Table 4. Contextual Factors in Interventions to Improve FIT/FOBT CRC Screening, stratified by setting26

Table 5. Implementation strategies used in interventions to improve FIT/FOBT CRC screening, stratified by setting33

Appendix A. Search Strategies

Database: Ovid MEDLINE(R) and Ovid OLDMEDLINE(R) <1946 to March Week 3 2015>; updated July 19, 2016

Search Strategy:

--------------------------------------------------------------------------------

1 exp Colorectal Neoplasms/ (152610)

2 exp Mass Screening/ (102379)

3 1 or 2 (249517)

4 exp Colonoscopy/ (22207)

5 exp Colonography, Computed Tomographic/ (1680)

6 exp Sigmoidoscopes/ (338)

7 (stool* adj test*).mp. [mp=title, abstract, original title, name of substance word, subject heading word, keyword heading word, protocol supplementary concept word, rare disease supplementary concept word, unique identifier] (427)

8 ((fecal* or feces or faecal$ or faeces) adj4 (immunochem* or immunohist*) adj4 test*).mp. [mp=title, abstract, original title, name of substance word, subject heading word, keyword heading word, protocol supplementary concept word, rare disease supplementary concept word, unique identifier] (434)

9 (fit and (fecal* or faecal$)).mp. [mp=title, abstract, original title, name of substance word, subject heading word, keyword heading word, protocol supplementary concept word, rare disease supplementary concept word, unique identifier] (307)

10 8 or 9 (584)

11 (fobt or ifobt).mp. (987)

12 occult blood.mp. (6015)

13 dna stool.mp. (58)

14 exp Polyps/ (26179)

15 exp Biopsy/ (228864)

16 14 and 15 (1271)

17 4 or 5 or 6 or 7 or 10 or 11 or 12 or 13 or 16 (29086)

18 3 and 17 (13521)

19 exp Colorectal Neoplasms/di, pa, pc, ra, ri, us (83132)

20 2 and 19 (5144)

21 18 or 20 (15222)

22 limit 21 to yr="1998 -Current" (11298)

23 exp Randomized Controlled Trial/ (387013)

24 exp Randomized Controlled Trials as Topic/ (97375)

25 randomized controlled trial.pt. (386986)

26 exp Single-Blind Method/ (20016)

27 exp Double-Blind Method/ (128196)

28 exp Random Allocation/ (82315)

29 23 or 24 or 25 or 26 or 27 or 28 (567475)

30 22 and 29 (869)

31 exp epidemiologic studies/ (1719501)

32 exp Evaluation Studies/ (200692)

33 longitudinal stud*.mp. (108357)

34 observational stud*.mp. (49593)

35 31 or 32 or 33 or 34 (1912760)

36 22 and 35 (3373)

37 limit 22 to "review articles" (1651)

38 30 or 36 or 37 (5439)

39 rural*.mp. (110463)

40 exp Health Services Accessibility/ (85603)

41 exp Healthcare Disparities/ (8135)

42 exp Health Status Disparities/ (8151)

43 exp Socioeconomic Factors/ (346765)

44 exp culture/ (116035)

45 39 or 40 or 41 or 42 or 43 or 44 (603150)

46 22 and 45 (710)

47 7 or 8 or 9 or 11 or 13 (1891)

48 22 and 47 (1201)

49 1 and 12 (3752)

50 48 or 49 (3902)

51 22 not (10 or 50) (8643)

52 exp "Continuity of Patient Care"/ (15487)

53 exp "diffusion of innovation"/ (15930)

54 exp Organizational Innovation/ (22962)

55 exp "Delivery of Health Care"/ (815714)

56 exp Professional Competence/ (87419)

57 exp "Costs and Cost Analysis"/ (185634)

58 52 or 53 or 54 or 55 or 56 or 57 (1001187)

59 50 and 58 (1311)

60 45 and 50 (353)

61 59 or 60 (1404)

62 22 and 58 (2610)

63 46 or 62 (2784)

64 63 not 61 (1644)

65 limit 50 to yr="1998 -Current" (2655)

***************************

The search strategy was modified for additional databases and searched on 4/09/2015 for the following dates:

- The Cochrane libraries (Central Register of Controlled Trials, Database of Systematic Reviews, and Database of Abstracts of Reviews of Effects)
- Scopus 1998-2009
- Scopus 2010-2015

Appendix B. Study Selection: Table of inclusion/Exclusion codes, definitions and key questions

| **Code** | **Definition** | **KQ1.** What is the effectiveness of various **interventions** to increase CRC screening with FIT/FOBT compared with other interventions or usual care in rural or low-income populations? | **KQ2**. How do **implementation strategies** (e.g., clinician champions, external facilitation) influence the effectiveness of interventions to increase FIT/FOBT screening for CRC in rural or low-income populations? | **KQ3.** How do **contextual factors** (e.g., patient, clinic, environmental features) influence the effectiveness of interventions to increase FIT/FOBT screening for CRC in rural or low-income populations? | **KQ4.** What are the **adverse effects** of interventions to increase FIT/FOBT screening for CRC in rural or low-income populations? |
| --- | --- | --- | --- | --- | --- |
| I–FIT  I–FOBT | Include: study meets all PICOTS criteria.  Specify test type and KQ(s) which the study addresses.  Specify study level, e.g., clinic, community, population | \| **Category** \| **Criteria** \| \| --- \| --- \| \| Population \| Adults aged 50-75 in rural, Medicaid, or socioeconomically disadvantaged populations in the U.S. who are eligible for CRC screening. \| \| \| Intervention \| Various strategies to improve CRC screening using Fecal Immunochemical Tests (FIT) or Fecal Occult Blood Tests (FOBT). Interventions may include one or more of the following and be implemented in the clinic, community, or a combination of these settings:   - Interventions to increase community demand: client reminders, client incentives, small media, mass media, group education, one-on-one education - Interventions to increase community access: reducing structural barriers, reducing client out-of-pocket costs - Interventions to increase provider delivery of screening services: provider assessment and feedback, provider incentives, provider reminders \| \| \| Comparator \| Usual care or other CRC screening interventions \| \| \| Outcome \| - Intermediate outcome: Change in rates of CRC screening using FIT/FOBT - Indicators of implementation success: Acceptability, adoption, sustainability of the actual intervention \| \| \| Time period \| 1998-present \| \| \| Setting \| Unrestricted \| \| \| Other criteria \| Admissible designs: systematic reviews, randomized controlled trials, including cluster randomized controlled trials; non-randomized trials, including interrupted time series studies with comparison groups; prospective and retrospective cohort studies; case-control studies; pre-post studies; and quasi-experimental studies that include a comparison group.  Language: We will include articles reported in English. \| \| | | | |
| X1 | Non-English publication (full-text article) | Include: Relevant English-language abstracts from non-English journals.  Exclude: Titles from non-English journals that provided no abstract in the systematic literature search. | | | |
| X2 | Not relevant in any way to CRC screening | --- | | | |
| X3 | Population not in scope (e.g., pediatric, obstetric, etc.) | Include: Studies that include rural or Medicaid populations of adults aged 50-75.  Exclude: Studies conducted outside of the US *(note common examples as they come up in the literature)* | | | |
| X4 | Study design or article type not in scope | Publication timeframe: 1998-present  Admissible designs: randomized controlled trials; non-randomized controlled trials, including interrupted time series studies with comparison groups; prospective and retrospective cohort studies; case-control studies; pre-post studies; and quasi-experimental studies that include a comparison group.  Exclude: Non-systematic or narrative reviews, opinions, case reports.  Also exclude studies that do not specify the modality used for CRC screening. | | | |
| X5 | Intervention not in scope. | Included interventions: Interventions at the clinic, community, or both levels to increase CRC screening using FIT/FOBT.  Excluded interventions:   - Colonoscopy, sigmoidoscopy. - Head-to-head comparisons of test usage without implementing an approach to improve uptake (i.e, no intervention was conducted; the study gathers descriptive usage data only). | | | |
| X6 | None of the reported outcomes are in scope | Included outcomes: Change in rates of CRC screening by modality (must include change in screening by FIT or FOBT);  Excluded outcomes: Change in rates of CRC screening only reported for all modalities. Patient intention to screen but not actual screening behavior. | | | |
| X7 | Other reason for exclusion – specify |  | *Note any patient or contextual factors that are not of interest* | *Note any implementation factors that are not of interest* |  |
| X8 | Duplicate publication | Article contains data that is similar to, and superceded by, a more complete or more recent publication. | | | |
| B | Background | Article meets one of the X codes above, but may be useful for background, methods, discussion, or referencing mining. | | | |

Appendix C. Quality Assessment

**Table C1. Quality Assessment for Randomized Controlled Trials (RTC) or Control Trial (CT) designs (N = 23).^a^**

| **Study** | ***Study design*** | | ***Cochrane criteria for assessing risk of bias (ROB)*** | | | | | | |
| --- | --- | --- | --- | --- | --- | --- | --- | --- | --- |
|  | **RCT** | **CT, not randomized** | **Randomization method adequately generated?** | **Allocation adequately concealed?** | **Knowledge of group assignment adequately prevented?** | **Incomplete outcome data adequately addressed?** | **Absence of selective outcome reporting?** | **Free of other potential biases?** | **Summary assessment High/Low/**  **Unclear ROB** |
| ***Clinic-based Studies*** | | | | | | | | | |
| Baker, 2014 [40] | 🗸 |  | Unclear | Yes. | Unclear | Yes | Yes | highly selected sample | Low |
| Coronado, 2011 [43] | 🗸 |  | Yes | Unclear | No, patients were not masked | Yes | Yes | Yes | Unclear |
| Coronado, 2014 [61] | 🗸 |  | Unclear | Unclear | No, patients were not masked | NA | Yes | No | Unclear |
| Davis, 2013 [44] | 🗸 |  | Unclear | Unclear | No; not blinded | Yes | Yes | Unclear | Unclear |
| Friedman, 2001 [45] | 🗸 |  | No (by days of the week) | No | Unclear | No (unable to address b/c study design) | Unclear | Unclear | High ROB |
| Goldberg, 2004 [46] |  | 🗸 | No; not randomized | Unclear | No for patients, outcome assessors; Yes for providers | Yes | Yes | Yes | Unclear |
| Goldman, 2015 [47] | 🗸 |  | Unclear | Yes. | Unclear | Yes | Yes | Unclear | Unclear |
| Greiner, 2014 [48] | 🗸 |  | Unclear | Unclear | Unclear | Yes | Yes | High refusal rate | Unclear |
| Gupta, 2013 [49] | 🗸 |  | Yes | Yes | Yes | Yes | Yes | Yes | Low |
| Hendren, 2014 [50] | 🗸 |  | Yes | Yes | Yes | Yes | Yes | Yes | Low |
| Jandorf, 2005 [51] | 🗸 |  | Unclear | Unclear | Yes | Yes | Yes | Unclear | Unclear |
| Jean-Jacques, 2012 [52] | 🗸 |  | Yes | Unclear | Yes | Yes | Yes | Yes | Low |
| Lasser, 2011 [53] | 🗸 |  | Yes | Unclear | Yes | Yes | Yes | Yes | Low |
| Lee, 2014 [87] | 🗸 |  | Unclear | Unclear | Unclear | Unclear: Self-report | Yes | Unclear | Unclear |
| Levy, 2013 [54, 55] | 🗸 |  | Unclear (method NR) | Unclear | Unclear | Unclear; only 62% followup | Yes | Unclear; low participation | Unclear |
| Potter, 2011 [56] | 🗸 |  | Unclear | Unclear | No: not blinded | Yes | Yes | Yes | Low |
| Potter, 2011 [66] |  | 🗸 | No: not randomized | N/A | No - not blinded | Yes | Yes | No: selection bias. | High |
| Roetzheim, 2004 [57] | 🗸 |  | Yes | Yes | Yes | Yes | Yes | Yes | Low |
| Tu, 2006 [60] | 🗸 |  | Yes | Unclear | No; patients were not blinded | Yes | Yes | High rates of refusal. | Unclear |
| ***Community-based Studies*** | | | | | | | | | |
| Braun, 2005 [41] | 🗸 |  | Yes | Unclear | No, not blinded | Yes | Unclear, self report. | Unclear: 64% were up to date | Unclear |
| Campbell, 2004 [42] | 🗸 |  | Unclear | Yes | No, subjects not blinded | Yes | Unclear, self report. | No: potentially uneven distribution of exposure. | Unclear |
| Thompson, 2006 [59] | 🗸 |  | Unclear | Unclear | Unclear | N/A. | Yes | Unclear | Unclear |
| ***Combined Clinic- and Community – based Studies*** | | | | | | | | | |
| Sarfaty, 2005 [62, 68] | 🗸 |  | N/A | N/A | N/A | Yes | Yes | Unclear | Unclear |

Abbreviations: CT = controlled trial, non-randomized; RCT = randomized controlled trial; ROB = risk of bias.

^a^ Studies assessed using the cochrane criteria for assessing risk of bias [25]. Four studies were excluded because they did not use a RCT or CT design. This included one clinic-based study [64], two community-based studies [63, 65] and one study that spanned clinic and community [67]. Findings for studies using a pre/post design appear in Table C2 [63-65]. One study was a feasibility study and did not fit the criteria for quality assessment tools [67].

**Table C2. Quality Assessment for Studies using a Pre-Post Design (N = 3).**

| **Criteria for pre-post studies [38]** | **Tu, 2014 [64] –**  **Clinic-based** | **Larkey, 2006 [63] –**  **Community-based** | **Wu, 2010 [65] – Community-based** |
| --- | --- | --- | --- |
| 1. Was the study question or objective clearly stated? | Yes | Yes | Yes |
| 2. Were eligibility/selection criteria for the study population prespecified and clearly described? | Yes | No | No |
| 3. Were the participants in the study representative of those who would be eligible for the test/service/intervention in the general or clinical population of interest? | Yes | Unclear; social networks were somewhat pre-established; sample potentially self-selected | Yes |
| 4. Were all eligible participants that met the prespecified entry criteria enrolled? | Yes | Unclear | No |
| 5. Was the sample size sufficiently large to provide confidence in the findings? | Yes | Unclear; no sample size calculation | Yes |
| 6. Was the test/service/intervention clearly described and delivered consistently across the study population? | Yes | Unclear | Unclear |
| 7. Were the outcome measures prespecified, clearly defined, valid, reliable, and assessed consistently across all study participants? | Yes | Unclear; measurement of FOBT use not specified, assumed self-report | Unclear |
| 8. Were the people assessing the outcomes blinded to the participants' exposures/interventions? | No | No | No |
| 9. Was the loss to follow-up after baseline 20% or less? Were those lost to follow-up accounted for in the analysis? | No (cross-sectional) | Yes | No |
| 10. Did the statistical methods examine changes in outcome measures from before to after the intervention? Were statistical tests done that provided p values for the pre-to-post changes? | Yes | No | No; different samples pre vs post |
| 11. Were outcome measures of interest taken multiple times before the intervention and multiple times after the intervention (i.e., did they use an interrupted time-series design)? | No | No | No |
| 12. If the intervention was conducted at a group level (e.g., a whole hospital, a community, etc.) did the statistical analysis take into account the use of individual-level data to determine effects at the group level? | Yes; adjusted for confounders | No | No |
| Summary assessment: High/Low/Unclear Risk of Bias | Low | High | High |

In each cell, enter: Yes/No/Other (CD = cannot determine; NA = not applicable; N = not reported)

Appendix D. DATA SUPplement

**Table 1. Characteristics and findings of included studies, stratified by intervention setting (clinic-based, community-based, or both)**

| **Study; Setting;**  **Target population** | **Screening test used; study funding source** | **Intervention arms** | **Follow-up duration;**  **Change in Screening Rates** | **Impact by Intervention Component** |
| --- | --- | --- | --- | --- |
| ***Clinic-based Studies*** | | | | |
| Baker, 2014 [40]  RCT  Urban Illinois (Chicago);  4 clinics affiliated with 1 FQHC system.  Mostly Latinos (89%) | FIT: Polymedco OC-Light Fecal Occult Blood Test  Funded by AHRQ | I (n=225): Stepped intervention: mailed FIT test, plus automated phone and text message reminder, plus 2nd reminder 2 weeks later for those who did not return FIT. Non-responders were contacted by a CRC screening navigator by phone, after 3 months.  C (n=225): Usual care included clinical computerized reminders, standing orders for MAs to give participants home FIT, and clinician feedback on CRC screening rates and using CRC screening as a quality metric for providers' incentive compensation formula. | 12 months  FIT completion within 6 months, I vs. C:  82.2% vs. 37.3% (*P* < .001)  Either FOBT or CS:  84.9% vs. 40.0% (*P* < .001)  By tier of Intervention:   - FIT completed prior to due date: 10.2% - FIT completed following mailing/initial call & text (before reminder call at 2 weeks): 39.6% - FIT completed after 2^nd^ reminder call/text: 47.8% of remaining 113 intervention patients - FIT completed after 3 month reminder call: 32% of 59 remaining intervention patients   Control/Usual Care:   - 11.1% completed FIT prior to due date | Patients who answered the automated call were more likely to complete FIT than those who did not have a completed automated call (51.2% vs 28.6%; P = .03), but equally likely to complete FIT vs. patients whose automated call was answered by voicemail (51.2% vs 42.4%; P = .22). Text message was not associated with FIT completion (44.3% vs 43.7%). Patients who spoke to the navigator at 3-month call were more likely to complete the FIT (50.0% vs 21.6%; P = .04). The results were similar in multivariate analyses that adjusted for demographics. |
| Coronado, 2011 [43]  RCT  Urban Washington (King County);  1 community clinic affiliated with an FQHC that specializes in care for Hispanic patients. | FOBT: NOS  Funded by Fred Hutchinson Cancer Research Center | I1 (n=168): Mailed FOBT with instructions I2 (n=168): Mailed FOBT plus outreach): home visits, telephone reminders, uses health promoters for home visits,  C (n=165): Usual care consisted of no formal prompting of colorectal cancer screening, other than what is provided during a physician visit | 9 months  FOBT screening: I1: Mailed FOBT only: 43 of 168 (26%, 95% CI 20 to 33)  I2: Mailed FOBT + outreach: 52 of 168 (31%, 95% CI 25 to 38)  C: 4 of 165 (2.4%, 95% CI, 0.9 to 6)  (P < .001 compared with usual care, both Tx). (P = .28 comparing I1 vs I2). | Substantial differences were noted in the proportions of respondents in the 3 groups who were considering having an FOBT in the next few months: 35% for the usual care group compared with 57% and 68% for the ‘‘mailed packet only’’ and the ‘‘mailed packet and outreach’’ groups, respectively. |
| Coronado, 2014 [61]  Non-randomized CT (pilot study)  Urban Oregon (Portland);  1 clinic affiliated with an FQHC system that operates a network of primary care clinics.  Latino (46%) and/or uninsured pts (59%) | FIT: OC Micro (PolyMedco, Inc, New York)  Funded by NIH Health Care Systems Research Collaboratory Demonstration Project; NIH/NCCAM | 3 clinics, one for each treatment arm.  I1 (n=197): “Auto”, Data-driven Electronic Health Record (EHR)-embedded program for mailing FIT kits; I2 (n=106): “Auto-Plus”, Mailed FIT kit plus live phone counselling and motivational interviewing techniques by the team’s bilingual Patient Care Coordinator; C (n=656): “Usual care”, A third safety-net clinic served as a passive control. 3-sample gFOBT cards (not FIT) were offered at clinic visits as part of usual care. | 6 months  FIT kits were completed by: I1: 44 of 112 in Auto (39.3%) I2: 37 of 101 in Auto Plus (36.6%)  C: FOBT was used by 7 of 656 in Usual care (1.1%).  Also reported CS use:  I1: 5 of 112 (4.5%)  I2: 3 of 101 (3.0%)  C: 5 of 656 (0.7%) | No differences in FIT completion between Auto and Auto Plus groups.  Authors note: preliminary estimates of effectiveness suggest that additional telephone-based outreach may not be needed. |
| Davis, 2013 [44]  RCT  Rural Louisiana;  8 clinics affiliated with 3 FQHCs; predominantly rural or low-income pts | FOBT: EZ Detect FOBT kit (Biomerica, Inc., Irvine, Calif)  Funded by NCI | I1 (n=282): Enhanced usual care plus brief education: pamphlet, video, and simplified FOBT instructions  I2 (n=404): I1, plus nurse support and follow-up.  C (n=275): Enhanced usual care: patients received a recommendation for CRC screening with FOBT kit | 12 months  % of patients who completed FOBT:  I1: 57.1%  I2: 60.6%  C (Enhanced usual care): 38.6%  P = .012, adjusted for age, race, sex, and literacy | Nurse support and education arms had significantly higher % FOBT completion within 12 months, compared with enhanced usual care. |
| Friedman, 2001 [45]  RCT  Urban Texas (Houston);  1 outpatient community clinic; Predominantly African American, female, low-income patients | FOBT: NOS  Funded by NR | I (n=110): Same as C, plus video of CRC education and FOBT techniques, watched prior to appointment. Video had peer educators (an older Afr Am woman and older Caucasian man) and a health professional (Afr Am female physician).  C (n=50): Participants received CRC brochure and questionnaire prior to clinic visit, FOBT kits after clinic visit and instructions. | 3 months  FOBT completed, I vs C:  43.6% vs. 36.0% (P = ns) | Authors noted a modest rate of compliance with FOBT screening in both I and C groups. |
| Goldberg, 2004 [46]  RCT  Urban Illinois (Chicago);  1 public hospital clinic that serves predominantly low-income African Americans | FOBT: NOS  Funding NR | I (n=59): Mailed FOBT cards with information and instructions with appointment reminder 2 weeks prior to a scheduled clinic visit  C (n=60): usual care; patients may receive FOBT cards directly from clinic physicians, subsequent to referral to the screening program by physicians, or may initiate screening without physician referral by stopping in the screening program office located near the waiting room. | 12 months after index appt  Time of return of FOBT cards,  I vs C.; OR (95% CI), P-value. At index appointment:  21/59 (35.6%) vs. 2/60 (3.3); 16.0 (3.5–71.4), P < .001 After the index appointment: 3/59 (5.1%) vs. 1/60 (1.7%); 3.2 (0.3–31.3), P = 0.36 During the year beginning from the index appointment: 24/59 (40.7%) vs. 3/60 (5.0%); 13.0 (3.6–45.5), P < 0.001 | The mailed FOBT kit was the only intervention. |
| Goldman, 2015 [47]  RCT  Urban Illinois (Chicago);  8 clinics affiliated with 1 FQHC system.  Mostly Latinos (78%) | FIT: OC-Light Fecal Occult Blood Test (Polymedco)  Funded by AHRQ | I (n=210): Patients had FIT kits mailed to their homes with provider letters and instructions. A few days after the mailing, study team sent an automated phone call, and 2 days later, a text message via a contracted commercial system. CRC Screening Navigator contacted them by phone 3 months later to non-responders.  C (n=210): Usual care included clinical computerized reminders, standing orders for MAs to give participants home FIT, and clinician feedback on CRC screening rates and using CRC screening as a quality metric for providers' incentive compensation formula. | 12 months  N (%) who completed FIT, I vs. C:  6 months: 77 (36.7%) vs. 31 (14.8%), p<0.001  12 months: 84 (40.0%) vs. 49 (23.3%), P < .001  N (%) who completed any CRC screening, I vs. C:  6 months: 77 (36.7%) vs. 32 (15.2%), p<0.001  12 months: 84 (40.0%) vs. 49 (23.3%), p<0.001 | N patients; FOBT completed (% of component): Mailing (p=0.10): Not returned 53 (29.8)  Returned 32 (15.6) Automated call:  Answered in person 36 (31.9) Answered by voicemail 21 (28.8), p=0.66 compared with in person Not completed 1 (4.2), p=0.006 compared with answered in person Text message (p=0.004) Completed 53 (32.3) Not completed 5 (10.9) Three-Month Call (n=152), FOBT completed between 13 and 26 Weeks (p=0.13): Spoke with patient 13 (16.5)  Unable to reach patient 6 (8.2) |
| Greiner, 2014 [48]  RCT  Urban Kansas (Wyandotte County);  9 safety net clinics affiliated with the County Safety-net Clinic Coalition; low-income pts | FIT: Enterix InSure™  Funded by NCI | All participants had an interactive, multimedia touchscreen computer session targeted to pt’s race/ethnicity, with narration (English or Spanish) through headphones. Costs for FIT and colonoscopy were covered by the study.  I (n=234): Computer session was on CRC screening; patients received questions on implementation intentions specific to the CRC screening test (FIT or CS) chosen by patient.  C (n=236): Generic health information on diet and exercise. | 6 months  Any CRC screening, I vs C:  54% vs 42%, OR 1.91 (95% CI 1.26, 2.89)  I: Of 126 that completed screening, 68 completed FIT and 58 completed CS.  C: Of 98 who completed screening, 51 completed FIT and 47 completed CS.  FIT vs. CS differences in completers were not significant between groups. | Exit interviews showed that 66% of study participants (309/468) talked to their doctor about CRC screening during their office visit after completion of touchscreen computer activities. For the 61% of all participants (249/408) that received a recommendation from their provider for CRC screening, majority received a recommendation for CS (54% CS, 13% FIT, 33% other). |
| Gupta, 2013 [49]  RCT  Urban Texas (Fort Worth & Tarrant County);  13 clinics affiliated with a hospital system.  Uninsured pts | FIT: OC-Auto FIT CHEK (Polymedco)  Funded by Cancer Prevention and Research Institute of Texas; University CTSA (NIH/NCRR funded CTSA); NIH/NCI | I1 (n=1593): FIT outreach. Simultaneously mailed no-cost FIT and postage paid return envelope. I2 (n=479): Colonoscopy outreach. Mailed invitation to schedule a no-cost colonoscopy. I3 (n= 3898): Usual care, consisted of opportunistic primary care visit-based screening  The FIT and colonoscopy outreach interventions both included a mailed English and Spanish invitation for free screening and information on risk for CRC based on age; 2 automated phone messages spaced 2 weeks apart; up to 2 live phone reminders to non-completers within 3 weeks of invitation; and aid with scheduling and instructions for colonoscopy. | 12 months  Screening within 1 year:  I1: FIT outreach: 40.7% (95%CI, 38.3%-43.1%)  I2: Colonoscopy outreach: 24.6% (95% CI, 20.8%-28.5%)  C: Usual care: 12.1% (95% CI, 11.1%-13.1%)  P < .001 for all comparisons | Within sex and race/ethnicity strata, both FIT and colonoscopy outreach approaches were superior to usual care for increasing screening. |
| Hendren, 2014 [50]  RCT  Urban New York (Rochester);  1 family practice.  Practice-level intervention;  low-income, and ethnic minority pts | Mixed FIT & FOBT: NOS  Funded by ACS | I (n=114): Patients received letters with CRC screening info offered phone assistance from outreach worker; FIT kits with 2^nd^ letter; automated phone calls to pts. Practice received point-of-care prompt sheets each week, containing reminder that pt was past due for mammography and/or CRC screening, for medical assistants to distribute at intervention-randomized patient appointment.  C (n=126): Usual care, NOS | 12 months  CRC screening, I vs. C: 37.7 vs. 16.7 (P = .0002)  Among those screened,  I (N=43) vs. C (N=21):  Used a FIT kit: 44% vs. 14% (19 vs 3 pts)  Used traditional FOBT kit:  12% vs. 33% (5 vs. 7 pts)  CS: 44% vs. 52% | Authors suggested that the mailed FIT kit portion of the intervention was particularly effective. |
| Jandorf, 2005 [51]  RCT  Urban New York (East Harlem);  1 clinic affiliated with 1 FQHC.  Low-income pts | FOBT: NOS  Funded by NCI | All patients received FOBT cards as well as a recommendation to undergo endoscopic screening from their physician.  I (n=38): Patient Navigator (PN) provided written reminders, telephone calls, and/or scheduling assistance, encouraging participation in CRC screening.  C (n=40): Usual care; all patients received FOBT cards as well as a recommendation to undergo endoscopic screening from their physician. | 6 months  I vs. C:  FOBT after 3 weeks (before navigation):  26.3 vs. 17.5% (P = ns)  FOBT after 3 months: 42.1 vs. 25.0%  (P = 0.086)  Endoscopy at 3 months: 15.8 vs. 5.0%, P= 0.115 (n.s.)  Endoscopy at 6 months: 23.7% vs. 5.0%, P= 0.019 | The PN group had increased completion of FOBT cards after 3 months, though not statistically significant.  Significantly more PN+ patients completed endoscopy at 6 months: 23.7 vs. 5.0% (P = 0.019).  Possible dilution of effect on FOBT: physicians were asked to recommend both FOBT and endoscopy to all patients. |
| Jean-Jacques, 2012 [52]  RCT  Urban Illinois (Chicago);  1 clinic affiliated with 1 FQHC.  Predominantly low income, uninsured, ethnic minorities | FOBT: Hemoccult II SENSA Elite kit (Beckman  Coulter, Inc)  Funded by NIH/NCRR; CTSA | I (n=104): Pts received letter that they were overdue for screening, CRC fact sheet and FOBT kit; those who did not return it within 2 wks received a scripted phone call from a bilingual (English/Spanish) lay health educator. Outreach included up to 3 call attempts each spaced 2 weeks apart. Patients were instructed to return kit to laboratory in person or in postage-paid envelope.  C (n=98): Pts could be referred for screening during usual clinic visits. | 4 months; 12 months post-hoc analysis  FOBT at 4 months, I vs. C:  29 vs. 4% (P < .001)  Any CRC screening at 12 months, I vs. C:  38 vs. 15% (P = .002) | Outreach led to significantly greater FOBT completion, however, 70% of patients assigned to the outreach arm did not complete CRC screening during the initial 4-month study period. |
| Lasser, 2011 [53]  RCT  Urban Massachusetts (Cambridge, Sommerville, & Everett);  6 PBRN affiliated clinics.  Multicultural, low-income population | FOBT: NOS  Funded by ACS | I (n=235): Patient received letter about overdue screening, CRC brochure in their language, and notice that a navigator would call them. Patient Navigator (PN) spent maximum of 6 hours in 6 months; included tailoring to the preferences of patients (FOBT cards mailed to patients or help with colonoscopy referral) and informed decision making. PN made up to 11 call attempts (leaving at least 2 messages) to each patient within a 3-week period.  C (n=230): Usual care, NOS | 12 months  CRC screening, I vs. C:  33.6% vs. 20.0% (P < .001)  FOBT: 7.2% vs 6.5 (P = .76).  Colonoscopy: 26.4% vs 13.0% (P < .001) | I-group pts whom navigators were able to contact were significantly more likely to be screened than those they unable to contact (39.8% vs 18.6%; P .001).  A larger proportion of I group pts received CS vs. controls (26.4% vs 13.0%; P .001).  FOBT completion was similar (7.2% vs 6.5%; P=.76). |
| Levy, 2012 [54]  Levy, 2013 [55]  RCT  Rural Iowa;  16 PBRN affiliated clinics.  Practice-level intervention.  98.4% Caucasian | FIT: Clearview ULTRA FOB FIT (Inverness, Waltham, MA)  Funded by ACS; University of Iowa, Dept of Family Medicine | I1 (n=185): Physician chart reminder I2 (n=186): I1, plus mailed packet of written and DVD educational materials, a refrigerator magnet to remind the subject about CRC screening, and a FIT with a postage-paid return envelope I3 (n=187): I1 + I2, plus a structured telephone call from project staff to provide education, assess interest in screening, explain the screening tests, address barriers and preferences, and encourage screening.  C (n=185): Usual care, NOS | Mean 15 months (range 13.6 to 19.3 months)  Any CRC test:  I1: 20.5% (38/185)  I2: 56.5% (105/186)  I3: 57.2% (107/187)  C: 17.8% (33/185)  P = .0001 for I2 and I3 vs. C.  FIT screening was the major contributor to screening in the mailed education groups:  I2: 84/186 (45.2%)  I3: 91/187 (48.7%) | CRC screening rate in mailed intervention groups (I2 and I3) was significantly higher vs. C.  No effect from the chart reminder vs. usual care (OR 1.2; 95% CI 0.7–2.0); and a significant effect from the mailed education/FIT (OR, 6.0; 95% CI, 3.7–9.6) and the mailed education/FIT plus phone call (OR, 6.2; 95% CI, 3.8 –9.9). |
| Potter, 2011 [56]  RCT  Urban California (San Francisco);  6 Public Health Clinics.  Marginally housed, homeless, Latinos, African Americans | FOBT: Hemoccult II (Beckman Coulter)  Funded by CDC; UCSF Clinical and Translational Science Institute (NIH/NCRR funded CTSA) | I (n=695): During intervention weeks, nurses routinely initiated the offering of FOBT to eligible patients who were given flu shots.  C (n=677): During control weeks, nurses provided FOBT with flu shot only when ordered by the primary care clinician during usual care. | 24 months  FOBT completion, I vs C: Pre: 148/604 (25%) vs 70/412 (17%) Post: 120/746 (16%) vs 47/514 (9%) Among patients not adherent to CRC screening at baseline:  FOBT completion, I vs C: 53/234 (22.6%) vs 24/167 (14.2%) The increase in FOBT adherence was marginally-significant (adjusted OR = 1.77; 95% CI 0.98, 3.18).  Any CRC adherence: 1.70 (1.05, 2.75) | Authors note: This study intervention was designed as a practical clinical trial that would produce results that would be relevant and replicable in routine clinical practice. Nurses were given autonomy in terms of how to offer FOBT. |
| Potter, 2011 [66]  Cohort study  Urban California (San Francisco);  1 Public Health Center (Chinatown Public Health Center).  Low-income Chinese Americans | FOBT: Hemoccult II (Beckman Coulter)  Funding NR | Flu shots are offered to all patients at all clinic visits seasonally.  I (n=970): Accepted flu shot. Shown 4-minute Cantonese language video and given FOBT kit, with instructions.  C (n=529): No flu shot given/flu shot refusers. | 6 months  FOBT:  PC visit c/flu shot = 38.4%  PC visit s/flu shot = 17.0%  (P < .001)  Adjusted OR of FOBT completion  comparing primary care visit c/flu shot vs. s/flu shot:  3.46 (95% CI 2.35 to 5.10) | Authors concluded that pairing of flu shots with FOBT was an effective intervention. No increase in FOBT was observed during flu shot period in the year prior to intervention. |
| Roetzheim, 2004 [57]  RCT  Rural Florida;  8 clinics participating in a county-funded health insurance plan; uninsured pts who do not qualify for Medicaid or Medicare | FOBT: NOS  Funded by NCI | I (n=596): Clinics received materials kit with a cancer-screening checklist to be completed by patients and chart stickers (red, yellow, and green) that indicated whether recommended screening tests had been ordered and completed.  C (n=600): Usual care, NOS | 12 months  % of patients who completed FOBT,  I vs. C: 40.1% vs. 11.9%  I change: 35.9% to 40.1%  C change: 22.1% to 11.9%  The odds of FOBT screening more than doubled: Multivariate OR 2.56 (95% CI, 1.65 to 4.01), P <.0001 | The authors remarked that the intervention’s low cost, no need for computers, and involvement of the patient in the screening process make it a novel approach. |
| Singal, 2015 [58]  RCT  Urban Texas (Dallas County);  12 community-based primary care clinics affiliated with a safety-net health system (publicly funded, integrated health system).49% Hispanic | I1: FIT (Polymedco OC-Auto FIT-CHEK)  C: FIT (Beckman Coulter Hemoccult ICT)  Funded by NIH/NCI | I1 (n=2400): FIT arm: pts received mailed letter on CRC risk with FIT kit. Research staff called non-responders within 2 weeks using a standardized script in English or Spanish; 3 attempts were made.  I2 (n=2400): Pts received mailed letter on CRC risk with invitation for CS and phone number for scheduling. Research staff called non-responders after 2 wks. Patients who called were triaged to open-access CS or pre-procedural review based on results of a structured history form; informed of $25 copay for CS; received CS prep materials by mail. Trained staff called patients 10 days and 2 days before the CS appointment to review prep procedure and answer questions.  C (n=1199): Usual care – received visit-based CRC screening at the discretion of their primary care providers | 12 months  CRC screening:  I1, FIT: Any = 58.8% (majority outreach FIT, 79.8%)  I2, CS: Any = 42.2% (half usual care FIT)  C: Any = 29.6% | Screening was significantly higher in FIT compared with CS (P<0.001). Both were significantly higher compared with usual care (P<0.001) |
| Tu, 2006 [60]  RCT  Urban Washington (Seattle);  1 community clinic serving predominantly limited English proficient Asian immigrants (International District Clinic).  Chinese patients | FOBT: NOS  Funded by NCI | I (n=105): A trilingual and bicultural health educator, bilingual materials (video, motivational pamphlet, informational pamphlet, FOBT instructions), and FOBT cards.  C (n=105): Usual care consisted of FOBT ordered by primary care providers. Medical assistants implemented all orders for FOBT and instructed patients to return completed FOBT cards to the clinic laboratory for processing and documentation of results on a laboratory form. | 6 months  FOBT, I vs C: 69.5% vs 27.6% Crude odds ratio: 5.98 (95% CI, 3.29 to 10.85) OR adjusted for age: 5.91 (95% CI, 3.25 to 10.75) | 77% of intervention patients elected to take the video home to review. The authors postulate that members of minority communities with limited health information are more likely to review health information (e.g., videos and pamphlets) provided to them either by clinic staff or by mail. |
| Tu, 2014 [64]  Pre-post, using cross-sectional data  Urban Washington (Seattle);  2 community clinics serving predominantly limited English proficient Asian immigrants.  Vietnamese and Chinese patients | FOBT: NOS  Funded by NCI | A quasi-experimental study examined CRC screening adherence before and after the 24-month intervention.  I (n=604): Small media (DVD and pamphlet) translated into Vietnamese from Chinese; medical assistants distributing the small media instead of a health educator; and presentations on CRC screening to the medical assistants.  C (n=412): Usual care: FOBT ordered by primary care providers; MA implemented all orders and instructed patients to return completed FOBT cards to clinic laboratory for processing or patients referred for colonoscopy. | 24 months  FOBT completion, I vs C: Pre: 148/604 (25%) vs 70/412 (17%) Post: 120/746 (16%) vs 47/514 (9%) Any CRC screening, I vs. C: Pre: 254/601 (42%) vs 158/412 (38%)  Among patients non-compliant at baseline:  FOBT completion, I vs C: 53/234 (22.6%) vs 24/167 (14.2%) The increase in FOBT adherence was marginally-statistically significant (adj OR = 1.77; 95% CI 0.98, 3.18).  Any CRC adherence: 1.70 (1.05, 2.75) | During the study period, both I and C clinics sites experienced a decrease in FOBT.  Clinic staff turnover during the study was high (90%) and potentially reduced intervention effectiveness.  Initially MAs at ICHS had greater cultural and linguistic congruence with their patients, an intended aspect of compatibility; staffing changes with certified MAs who were also proficient with EMRs reduced cultural and linguistic congruence with patients. |
| ***Community-based Studies*** | | | | |
| Braun, 2005 [41]  RCT  Hawaii;  16 civic clubs.  Native Hawaiians | FOBT: NOS  Funded by NR | I (n=69): In addition to Control procedure, SLT-Native Hawaiian CRC survivor told personal story; Native Hawaiian physician gave educational presentation and FOBT instructions; attendees encouraged to share info with family members to request FOBT kits for themselves; multiple phone call reminders.  C (n=52): culturally tailored educational material, FOBT kit, single reminder call. Non-Hawaiian nurse gave educational presentation. | 16 weeks  Pre % who were up to date with CRC screening at baseline (% who had FOBT in past year);  Post % who completed free FOBT kit during study:  I: Pre 59% (FOBT 30%); Post FOBT 33%  C: Pre 69% (FOBT 39%); Post FOBT 40% | Authors observed: the culturally targeted presentation worked well in both arms, regardless of who presented it (native Hawaiian vs. non-Hawaiian nurse). Authors speculated that the SLT components of the family-member challenge and the multiple reminder phone calls made the Tx arm too invasive and burdensome, leading some to passively resist calls for screening. |
| Campbell, 2004 [42]  RCT  Rural North Carolina (5 eastern counties);  12 churches each with 80+ active members.  African American church members | FOBT: NOS  Funded by ACS; US Department of Agriculture; NIH | I1 (n=51): LHA arm using "natural" social networks. Churches were asked to identify potential lay advisors among their congregation, who were then invited to participate in LHA training. LHAs provided CRC information through existing networks; organized and conducted 3+ church-wide activities.  I2 (n=76): TPV arm: individual computerized tailoring for health promotion, included 4 personalized computer-tailored newsletters and 4 targeted videotapes mailed to participants' homes bimonthly for 6 months.  I3 (n=87): Combined group: received TPV and LHA  C (n=69): Control churches were offered 2 health education sessions and speakers on topics of their choice (among HIV/AIDS, adolescent health, child care and health, prostate cancer awareness, and elderly health issues). | 1 year after baseline survey completion, approx. 3 months after intervention  % FOBT in past year, Baseline / followup:  I1, LHA: 23.5 / 33.3  I2, TPV: 19.7 / 36.8  I3, LHA+TPV: 19.5 / 31.0  C: 30.4 / 21.7  (P = .08; Determined by logistic regression models controlling for gender, age, and baseline values) | At the 1-year follow-up, all groups showed improvement in FOBT rates, however the TPV-only group had the largest increase in the proportion of participants who had FOBT: 87% increase over baseline.  A multicomponent approach combining a tailored and a targeted home-based intervention with a lay helping, church-based was not more effective than either intervention alone. |
| Larkey, 2006 [63]  Pre-post study  Urban Arizona (Phoenix);  Churches and community-based organizations NOS.  Hispanic women | FOBT: NOS  Funded by St. Luke’s Charitable Health Trust, Phoenix, Arizona | Single group (n=186. Las Mujeres Saludables/Healthy Women): a culturally aligned education program using community health advisors and emphasizing social support, used a Promotora-led community classroom format with methods for developing group identity, cohesion, and social support. | 3 months  Of 169 non-compliant at baseline, 7 individuals (4.1%) completed FOBT w/in 3 month period. | Authors note that the potential for evaluating the effects of the intervention on CRC screening was likely limited by the short timeframe (3-months). |
| Thompson, 2006 [59]  RCT, used cross-sectional pre-post data  Rural Washington (Yakima Valley);  20 agricultural communities.  Hispanic agricultural workers | FOBT: NOS  Funded by NCI | Total n=1,962  I: 10 communities received a comprehensive approach to cancer prevention over 30 months, with activities directed at the community, organizational, small group, and individual levels; included health fairs, block parties, festivals, fun runs, and other events; activities at churches and schools; home health parties; outreach by promotoras.  C2: 10 communities matched on size, proportion Hispanic, and presence of clinic received no intervention.  C2: 3 communities that were geographically distant received no intervention. | 30 months  Analysis includes 823 aged 50+ non-compliant at baseline: 384 vs. 439.  I vs. C:  Self report; adjusted for community baseline ethnic-specific rates:  among those never screened prior:  Hispanic: 57.1 vs 48.3 (P = ns)  White: 47.4 vs. 29.0 (P = .03)  Among those who had prior screening but not compliant at baseline:  Hispanic: 78.5 vs. 64.0 (P = ns)  White: 62.2 vs. 47.8 (P = ns)  % never screened who received CS during study:  Hispanic: 92.6 vs. 86.1 , p= 0.55  White: 64.8 vs. 54.3, p= 0.21 | NR |
| Wu, 2010 [65]  Pre-post study  Urban Michigan (University of Michigan);  University of Michigan’s Healthy Asian Americans Project.  Asian Americans | FOBT: Colo-Scan by StarLine  Funded by Michigan Department of Community Health | Total n=304. Expanded established health promotion program to include community-based CRC education. Hired local community coordinator to outreach to community gathering places. To increase awareness of CRC, we used the various Asian language media (newspapers, posters) and health fairs; participants were recruited to attend an educational seminar on early detection of CRC. An FOBT kit was offered for purchase at $2 or less. | 1 year  Self-report at 12 months, among 156 who could be reached (of 304 at baseline):  Any screening: 78% vs 33%  FOBT: 70 (45%)  Colonoscopy: 45 (29%)  Flexible sigmoidoscopy: 2 (1%)  Both FOBT and sigmoidoscopy: 4 (2%) | After the intervention, the percentage giving the correct answer increased for each knowledge scale item, indicating that the seminar covered the range of intended topics and attention, was retained throughout. The paired t tests showed that the increase was statistically significant (p<0.05) for seven of the 10 items. |
| ***Combined Clinic- and Community-based Studies*** | | | | |
| Redwood, 2011 [67]  Feasibility assessment  Urban Alaska (Anchorage);  Anchorage Neighborhood Health Center and FQHC and Alaska’s largest community health center.  Low-income, underinsured, or uninsured patients | FIT: NOS  Funded by Ride for Life Alaska | Total n=549. Ride for Life (RFL) began as a CRC screening fundraiser, then formed a partnership with ANHC to promote CRC screening among low-income and underin­sured ANHC patients. RFL and ANHC engaged gastroenterologists, medical practices, and pathology services to contribute pro bono and reduced-fee services for CRC screening. The program was promoted via signs in the clinic and exam rooms, flyers mailed to patients, on ANHC website, and local radio programs. Eligible patients were mailed an annual postcard during Colorectal Cancer Awareness Month (March) inviting them to pick up a free iFOBT kit at the clinic. | 1 year  72% during first year (2007)  During 2007-2010, there were 2,561 FOBT given to patients, and 1,558 were completed (61%) | Authors stated that the presence of a dedicated screening care coordinator based at ANHC is a necessary component of the program; this person is a liaison between the medical community members donating their services and patients in need of screening. After the creation of this position via funding by RFL in 2008, screening numbers under the program increased substantially. |
| Sarfaty, 2005 [62]  Sarfaty, 2006 [68]  Non randomized CT  Urban Maryland (Montgomery County);  County-wide program.  Low-income, uninsured, ethnically diverse population | FOBT: NOS  Funding NR | 1672 individuals registered for a clinical screening program with a registered nurse who provided education, made colonoscopy referrals, and provided case management.  I1 (n=375): Referred Group  I2 (n=936): Educator Group  I3 (n=361): Telephone Group responding to media outreach.  Individuals registered via a cancer telephone line, a session with an educator, or direct physician referral. Education and outreach included advertising and community-based direct education conducted by health educators employed by multiple partners. | 2.5 years (6 months after 2-year registration period)  Any screening (CS, FS, FOBT): 52%  FOBT kits were returned by 334 (58.5%) of 571 distributed.  Of 32 positive FOBT results, 24 (75%) were followed up by colonoscopy. | Individuals registered for the clinical screening via a cancer telephone line, a session with an educator, or direct physician referral.  % of group that completed FOBT (% CS):  Referred group: 8.8% (52% CS)  Educator group: 29.1% (15% CS)  Telephone group: 7.8% (53% CS) |

Abbreviations: ACS=American Cancer Society; C = Control; CRC=Colorectal Cancer; I1 = Intervention Arm 1; I2 = Intervention Arm 2, etc.; LHA=lay health advisor; NCCAM=National Center for Complementary & Alternative Medicine; NCI=National Cancer Institute; NCRR=National Center for Research Resources; TPV=tailored print and video.

**Table 2. Participant recruitment and recruitment success in intervention studies to improve FIT/FOBT screening for CRC, stratified by intervention setting (clinic-based, community-based, or both)**

| **Study,**  **Design** | **Patient Eligibility and Recruitment** | **IRB approval;**  **Informed consent or waiver** | **Recruitment Success** |
| --- | --- | --- | --- |
| ***Clinic-based Studies*** | | | |
| Baker, 2014 [40]  RCT | EHR data was used to identify eligible patients: aged 51 to 75 years; preferred language English or Spanish; and previously completed FOBT with negative result. | IRB approved; waiver of informed consent. | 9 patients had the home FIT returned for invalid addresses  171: had an automated call completed; 86 were answered in person, and 85 were answered by voicemail.  Over half of eligible patients received a text message |
| Coronado, 2011 [43]  RCT | All Hispanic patients aged 50 to 79 years who had a clinic visit at the South Park clinic from January 1, 2002 through May 31, 2006 were drawn from the computerized clinic records system. Recruitment not described. | IRB/consent process NR | Of 336 FOBT packets mailed, 289 (86%) were received.  Among the 168 patients in the Mailed FOBT + Outreach group, 115 (68%) could be reached at the phone number on record, and 13 (7.7%) completed a home visit. |
| Coronado, 2014 [61]  Non-randomized CT | Patients aged 50–74 whose primary language was English or Spanish, who were not up-to-date with CRCS, who received care in the past year at either of the two participating clinics. Eligible patients were sent an introductory letter. | IRB approval NR.  Patients sent mailed letter with opportunity to opt out of study. | FIT kit was mailed to: I1: 109 of 112 in Auto (97.3%) I2: 97 of 101 in Auto Plus (96.0%)  Of 66 Auto Plus pts identified for theory-based phone counseling, 8% of those identified (and 17% of those successfully reached) returned their FIT kits. |
| Davis, 2013 [44] RCT | MAs identified potentially eligible patients during clinic visits by looking at age in chart (range: 50-85 years old) and asked patients if they would talk to an onsite research assistant about participating in a study on CRC prior to physician encounter. Interested patients were screened by the RA for eligibility: 1) English speaking, 2) current clinic patient, 3) average risk for CRC, 4) not up to date, and 5) not having acute medical concern. | IRB approved; informed consent | 961 (91.1%) of 1055 patients who met age criteria were eligible and enrolled.  61 (5.8%) were up to date with CRC screening and were excluded.  33 patients (3.1%) refused participation. |
| Friedman, 2001 [45]  RCT | Patients recruited from clinic waiting room.  Eligibility: 50 years or older, no FOBT in past 12 months, no personal history of CRC, English speaking, could provide written informed consent. | IRB consent process NR | No information about patients who did not participate is available. |
| Goldberg, 2004 [46]  RCT | A 50% random sample of age-eligible patients in the General Medicine Clinic Improvement Project registry was cross-linked with the electronic database of the Colon Cancer Screening Program. These patients were then crosslinked with the appointment system to select patients scheduled on a Tuesday afternoon in May or June 200. Patients with odd medical record numbers assigned to intervention group, patients with even medical record numbers to usual care group. | QI project, not subject to IRB review.  Patients did not sign consent at time of registry development or study initiation. | 78% attended the clinic on the date of the index appointment in the intervention group, and 75% attended the clinic in the control group. Seven (12%) of the letters sent to the intervention group were returned by the post office (unable to deliver). |
| Goldman, 2015 [47]  RCT | An EFHC programmer queried the EHR to identify eligible patients aged 50–75, who had a preferred language of English or Spanish, at least two EFHC visits over 2 years before the study, and no documentation of CRC screening. | IRB approved; waiver of informed consent. | Total N (%) Mailing:  Not returned 178 (84.8)  Returned 32 (15.2) Automated call:  Answered in person 113 (53.8) Answered by voicemail 73 (34.8), p=0.66 Not completed 24 (11.4) p=0.006 Text message (p=0.004): Completed 164 (78.1) Not completed 46 (21.9)  Three-Month Call (n=152):  Spoke with patient 79 (52.0)  Unable to reach patient 73 (48.0) |
| Greiner, 2014 [48]  RCT | Health center staff from each clinic referred patients to kiosks in clinic waiting areas where RAs were available to facilitate eligibility screenings.  Eligibility: patients > 50 years old with a provider visit the day of enrollment.  Exclusions: Income of >150% of the FPL, lacked a home address and working telephone, were up-to-date with screening, were at above average risk, had history of polyps or CRC, other household members in the study, or had a cognitive impairment. | IRB approved; informed consent | Computer interface: Almost all participants (99%) reported that they understood the messages they received on the touchscreen computer. Almost all felt that they received all the information they needed (97%). |
| Gupta, 2013 [49]  RCT | We included men and women, aged 54-64 years, who were uninsured but enrolled in the JPS medical assistance program. We excluded patients meeting 1 or more of the following criteria: 1) up-to-date CRC screening, 2) no address or phone number on file, 3) primary language other than English or Spanish, 4) history of CRC, inflammatory bowel disease, or colorectal polyps, 5) no recent health system visits (any visit within 8-month period prior to randomization, or 6) incarceration. | IRB approved; waiver of informed consent. | All randomized Ss received the allocated intervention |
| Hendren, 2014 [50]  RCT | An electronic listing of all active patients was obtained, and eligibility criteria for patient inclusion were reviewed in the practice’s EHR. | IRB approved; waiver of informed consent | The automated phone calls were answered in most cases; out of 670 calls to 183 subjects, 86 % of calls were “successful” and 96%of subjects received at least one “successful” call.  Of 92 FIT kits mailed, 19 (20.7%) were successfully used. |
| Jandorf, 2005 [51]  RCT | Participants were identified from among patients attending a primary care practice in east Harlem, NYC between January and May 2002. Eligible subjects included men and women aged 50 and older who were not up to date. Charts of all scheduled patients were reviewed for eligibility, the RA approached prospective participants. A 17-item sociodemographic questionnaire was also completed at recruitment. | IRB approved, informed consent. | One hundred twenty-five people were approached, of whom 88 (70%) agreed to participate |
| Jean-Jacques, 2012 [52]  RCT | Patients were eligible for this study if they were adults aged 50-80 years who had at least 2 visits to the study site between July 1, 2008-December 31, 2009, with no history of CRC or total colectomy, and with no documented FOBT within 1 year, sigmoidoscopy within 5 years, or colonoscopy within 10 years. | IRB approved; waiver of informed consent. | The outreach coordinator attempted to call the 103 pts who did not return FOBT kit within 2 wks of 1^st^ mailing. 40% of pts were reached within 3 phone call attempts: 25 on the 1st call, 12 on the 2nd call, and 4 on the 3rd call. 23% had phone numbers that were wrong or not in service. 15 of the 30 completed FOBT kits were returned within 6 wks of the 1st mailing.  A 2nd FOBT kit was mailed to the 57 patients (55% of Tx group) who had not been reached within 3 call attempts and had not returned an FOBT kit within 6 wks of the initial mailing. |
| Lasser, 2011 [53]  RCT | We included a sample of patients aged 52-74 years who had had 1 visit to a PCP in each of the 2 previous years, had not completed CRC screening according to USPSTF guidelines and who spoke English, Haitian Creole, Portuguese, or Spanish as their primary language. We used the EHR to identify patients who were not-up-to-date. Patients were excluded if they had acute illness, an end-stage medical disease, severe psychiatric condition, active substance abuse, or cognitive impairment. | IRB approved, consent waived. | 235 randomized to Tx group:  181 (77%) Contacted by navigator  54 (23%) Not reached by navigator |
| Levy, 2012 [54]  Levy, 2013 [55]  RCT | Random sample of patients sent letter to participate in study. Participants recruited using Dillman technique, which included pre-notice letter with a $2 bill followed by a full packet of materials 2 weeks later. Reminders sent and follow-up calls made to non-responders.  Only individuals due for CRC screening were eligible for intervention. Individuals with a family history of colon cancer, ulcerative colitis, Crohn’s disease, or personal history of CRC were NOT excluded. | IRB approved; informed consent | 8,372 patients were invited to participate. Baseline questionnaires were returned by 2008 (24%); 743 patients (9% or 8,372 and 24% of 2008) were eligible to participate.  Of all patients returning questionnaires 42% (838/2008) were due for CRC screening based on self-report. |
| Potter, 2011 [56]  RCT | Patients aged 50 –75 years who received FLU during primary care visits during an 18-week intervention at six community clinics in San Francisco. Nurses determined whether CRC screening was due by consulting either electronic or paper-based medical records. | IRB approved; consent process NR. | A total of 3600 patients aged 50–75 came in for at least one primary care visit during the 18-week study period. Of these, 1372 patients were documented in the electronic medical record as having FLU during one of the visits. A total of 695 of these patients came in for primary care on a FLU–FOBT date, and the remaining 677 came in for primary care on a FLU-only date. |
| Potter, 2011 [66]  Cohort study | Patients at a community clinic serving low-income Chinese Americans who came in for a primary care visit during the time when flu shots were available and were eligible for CRC screening. Nursing staff offered flu shots to all eligible patients; those who agree are offered FOBT kits and shown a video on CRC. | IRB approved; consent process NR. | 970 (64.7%) of 1499 patients eligible for CRCS received flu shots + FOBT intervention. |
| Roetzheim, 2004 [57]  RCT | Clinics eligible if they (1) provided primary medical care 5 days a week, (2) majority of clinician providers agreed to participate, and (3) clinic was expected to continue operating in same fashion for following 24 months). 8 of 16 clinics not eligible; 8 remaining clinics randomized.  Cancer screening checklist completed by patients in clinic indicating if they were due for screening. Patient’s records were eligible for abstraction if (1) patient aged 50-75 years of age and (2) was an established patient – had at least 1 visit 12 months or more before the sampled visit. Additionally, patients with a personal history of colon cancer and those receiving colonoscopy or double-contrast barium enema in last 10 years were excluded from analysis. | IRB approved; waiver of informed consent | NR.  Data abstracted from charts of independent samples collected at baseline (n = 1,196) and at a 12-month follow-up (n = 1,237) was used to assess whether the patient was up-to-date. |
| Singal 2015  RCT | Patients aged 50-64 with at least 1 visit to a PHHS safety-net primary care clinic within the past year, who were not in compliance with CRC screening.  Patients excluded if they had (1) no address or telephone number on file, (2) spoke a primary language other than English or Spanish, (3) had a history of CRC, inflammatory bowel disease, colorectal polyps, or prior colectomy; and (4) incarceration. | IRB approved, consent waived. | 5999 randomized to 3 groups (2400 to CS, 2044 to FIT, 1199 to usual care)  100% of randomized subjects received the allocated intervention. |
| Tu, 2006 [60]  RCT | Investigators identified Chinese patients aged 50–78 years of age and spoke Cantonese, Mandarin, and/or English, using the ICHS database. A bilingual letter, in Chinese and English, from the medical director was mailed eligible patients to introduce the study. | IRB approved; informed consent not required.  Patients were given the option to decline participation. | 77% of intervention patients elected to take the video home to review. |
| Tu, 2014 [64]  Pre-post, using cross-sectional data | Vietnamese patients aged 51 to 76 actively attending International Community Health Services (ICHS), a community health center serving predominantly limited English-proficient (LEP) Asian immigrants. Study sample was identified from EMR review; recruitment not described. | IRB approved; consent process NR. | NR |
| ***Community-based Studies*** | | | |
| Braun, 2005 [41]  RCT | A previous study identified 56 civic clubs in Hawaii and conducted focus groups and surveys to examine CRC screening behaviors. The civic clubs were asked to volunteer for the current study.  Patients over 50 years old in each club considered eligible for CRC intervention. | IRB approved; informed consent. | 16 of 39 Hawaiian civic clubs participated. Each presentation was attended by 3-20 individuals aged 50 or older. |
| Campbell, 2004 [42]  RCT | 51 churches contacted by study team to determine eligibility (>80 active members) and assess interest in participation; 51 did not meet size criterion. Study packet sent to 26 remaining churches – research team met with pastor and interested church members who voted on participation. 10 churches were recruited from 26 approached and 2 additional churches added from master list (12 total).  All active members (i.e., those attending the study church at least 1 time per month), aged 18 or older, were considered eligible to participate. | IRB/Consent process NR | Of 1,463 eligible, 850 (58%) completed the baseline interview, 239. The overall baseline response rate was 66%, defined as the number of respondents divided by the estimated number of those eligible in the sample. The final sample for comprised 587 individuals who completed both the baseline and follow-up surveys, yielding a 72% response rate, with no significant differences between groups. |
| Larkey, 2006 [63]  Pre-post study | Promotoras used networks of contacts in churches and community centers to recruit participants (targeting low-income Hispanic women) over 9 months with staggered class start dates. | IRB/Consent process NR | 80.8% of 457 enrolled completed the class and completed post-intervention surveys. 186 were aged 50+ and eligible for CRC screening. |
| Thompson, 2006 [59]  RCT | Investigators identified and developed a partnership with 20 communities heavily populated by Hispanic agricultural workers. | IRB approved; consent process NR. | The response rate of the known eligible households where an adult was present(n = 2,083) was  83.1% at baseline,  89.4% for final survey. |
| Wu, 2010 [65]  Pre-post study | An established Healthy Asian Americans Project (HAAP) used community health fairs to recruit Asian Americans. HAAP coordinators conducted outreach to churches, temples, and civic organizations, posted announcements in Asian grocery stores, and in Asian language media. | IRB approved; consent process NR. | At 12-month follow-up, 156 (51.3%) of 304 participants could be reached for follow-up. |
| ***Combined Clinic- and Community-based Studies*** | | | |
| Redwood, 2011 [67]  Feasibility assessment | An annual fundraiser (Ride for Life) program partners with ANHC, Alaska’s largest community health center, to promote CRC screening and outreach to an ethnically diverse group of low-income underinsured or uninsured patients in Anchorage. The program performs outreach to patients via radio programs, websites, and flyers. | IRB/Consent process NR | Number of eligible patients not reported. |
| Sarfaty, 2005 #1742 [62]  Sarfaty, 2006 [68]  Non randomized CT | Montgomery County residents were recruited via outreach by media campaigns in English and Spanish; a bilingual phone line; and education sessions in healthcare settings, workplaces, and neighborhoods. County residents enrolled by physician referral, after contact with a health educator, or via telephone. | IRB/Consent process NR | FOBT kits were distributed to 571 (34%) of 1672 registered for the cancer screening program |

**Table 3. Intervention Components – Tracked for the most complex intervention arm tested in a study**

|  | Increasing Community Demand | | | | | | Increasing Community Access | | | | | | Increasing Provider Delivery | | | Misc | | Total Intervention Components | |
| --- | --- | --- | --- | --- | --- | --- | --- | --- | --- | --- | --- | --- | --- | --- | --- | --- | --- | --- | --- |
|  |  |  |  |  |  |  | Reducing Structural Barriers | | | | | Reducing out-of-pocket costs |  |  |  |  |  |  |  |
| **Reference;**  **Study design** | Client Reminder or Recall | Client Incentives | Small media | Mass Media | Group Education | One-on-One education | Provider Ordered In-Clinic Kit distribution | Systematic In-Clinic Kit Distribution | Direct Mail (FIT/FOBT mailed to participant home) | Pre-Addressed Stamped Envelope Provided | FIT/FOBT available by participant request |  | Provider Assessment & Feedback | Provider Incentives | Provider Reminder & Recall Systems | Patient Navigators | Other | MOST COMPLEX ARM | CONTROL/COMPARISON ARM |
| ***Clinic-based Studies*** | | | | | | | | | | | | | | | | | | | |
| Baker, 2014 [40]  RCT | 🗸 |  |  |  |  |  | 🗸 | 🗸 | 🗸 | 🗸 |  |  | 🗸 | 🗸 | 🗸 | 🗸 | 🗸^a^ | 10 | 5 |
| Coronado, 2011 [43]  RCT | 🗸 |  | 🗸 |  |  | 🗸 | 🗸 |  | 🗸 | 🗸 |  |  |  |  |  |  | 🗸^a^ | 7 | 1 |
| Coronado, 2014 [61]  Non-randomized CT | 🗸 |  |  |  |  |  | 🗸 |  | 🗸 | 🗸 |  |  |  |  |  | 🗸 | 🗸^a^ | 6 | 1 |
| Davis, 2013 [44] RCT | 🗸 |  | 🗸 |  |  | 🗸 |  | 🗸 |  | 🗸 |  |  |  |  |  | 🗸 | 🗸^b^ | 7 | 4 |
| Friedman, 2001 [45]  RCT |  |  | 🗸^c^ |  |  | 🗸 |  | 🗸 |  |  |  |  |  |  |  |  | 🗸^b^ | 4 | 4 |
| Goldberg, 2004 [46]  RCT | 🗸 |  | 🗸 |  |  |  | 🗸 |  | 🗸 |  | 🗸 |  |  |  |  |  |  | 5 | 2 |
| Goldman, 2015 [47]  RCT | 🗸 |  |  |  |  |  | 🗸 | 🗸 | 🗸 |  |  |  | 🗸 | 🗸 |  | 🗸 |  | 7 | 4 |
| Greiner, 2014 [48]  RCT |  |  | 🗸^d^ |  |  | 🗸 |  | 🗸 |  |  |  | 🗸 |  |  |  |  | 🗸^b^ | 5 | 4 |
| Gupta, 2013 [49]  RCT | 🗸 |  |  |  |  |  | 🗸 |  | 🗸 | 🗸 |  |  |  |  |  | 🗸 |  | 5 | 1 |
| Hendren, 2014 [50]  RCT | 🗸 |  |  |  |  |  |  |  | 🗸 |  |  |  |  |  | 🗸 |  |  | 3 | N/A |
| Jandorf, 2005 [51]  RCT | 🗸 |  |  |  |  | 🗸 |  | 🗸 |  |  |  |  |  |  |  | 🗸 |  | 4 | 1 |
| Jean-Jacques, 2012 [52]  RCT | 🗸 |  | 🗸 |  |  |  | 🗸 |  | 🗸 | 🗸 |  |  |  |  |  | 🗸 |  | 6 | 1 |
| Lasser, 2011 [53]  RCT | 🗸 |  | 🗸 |  |  | 🗸 |  |  |  |  | 🗸 |  |  |  |  | 🗸 |  | 5 | N/A |
| Levy, 2012 [54]  Levy, 2013 [55]  RCT | 🗸 |  | 🗸 |  |  | 🗸 |  |  | 🗸 | 🗸 |  |  |  |  | 🗸 | 🗸 |  | 7 | N/A |
| Potter, 2011 [56]  RCT |  |  |  |  |  |  |  | 🗸^e^ |  | 🗸 |  |  |  |  |  |  |  | 2 | 1 |
| Potter, 2011 [66]  Cohort study |  |  | 🗸 |  |  |  |  | 🗸 |  | 🗸 |  |  |  |  |  |  | 🗸^a^ | 4 | 1 |
| Roetzheim, 2004 [57]  RCT |  |  |  |  |  |  |  |  |  |  |  |  | 🗸 |  | 🗸 |  | 🗸^b^ | 3 | N/A |
| Singal, 2015  RCT | 🗸 |  | 🗸 |  |  | 🗸 | 🗸 |  | 🗸 | 🗸 |  |  |  |  |  |  | 🗸^a^ | 7 | 1 |
| Tu, 2006 [60]  RCT |  |  | 🗸 |  |  | 🗸 | 🗸 | 🗸 |  | 🗸 |  |  |  |  |  |  | 🗸^a^ | 6 | 2 |
| Tu, 2014 [64]  Pre-post, using cross-sectional data |  |  | 🗸 |  |  |  | 🗸 |  |  |  |  |  |  |  |  |  | 🗸^a^ | 3 | 2 |
| ***Community-based Studies*** | | | | | | | | | | | | | | | | | | | |
| Braun, 2005 [41]  RCT | 🗸 |  | 🗸 |  | 🗸 | 🗸 |  | 🗸 |  |  |  |  |  |  |  |  | 🗸^b^  🗸^f^ | 7 | 6 |
| Campbell, 2004 [42]  RCT |  |  | 🗸 |  | 🗸 | 🗸 |  |  |  |  |  |  |  |  |  |  | 🗸^f^ | 4 | 0 |
| Larkey, 2006 [63]  Pre-post study |  |  |  |  | 🗸 |  |  |  |  |  |  |  |  |  |  |  | 🗸^f^ | 2 | N/A |
| Thompson, 2006 [59]  RCT |  |  | 🗸 |  | 🗸 | 🗸 | 🗸 |  |  |  | 🗸 |  |  | 🗸 |  |  | 🗸^b^ 🗸^f^ | 8 | N/A |
| Wu, 2010 [65]  Pre-post study |  |  |  |  | 🗸 | 🗸 |  | 🗸 |  | 🗸 |  | 🗸 |  |  |  | 🗸 |  | 6 | N/A |
| **Combined Clinic- and Community-based Studies** | | | | | | | | | | | | | | | | | | | |
| Redwood, 2011 [67]  Feasibility assessment | 🗸 | 🗸 | 🗸 | 🗸 |  |  |  |  |  |  | 🗸 | 🗸 |  |  |  | 🗸 |  | 7 | N/A |
| Sarfaty, 2005 #1742 [62]  Sarfaty, 2006 [68]  Non randomized CT | 🗸 |  | 🗸 |  | 🗸 | 🗸 |  | 🗸 |  |  |  | 🗸 |  |  |  | 🗸 |  | 7 | 5 |

^a^ Interventions that provided materials that were tailored for specific cultures and/or were for low literacy

^b^ Studies included the completion of patient questionnaires or surveys about CRC screening knowledge and behaviors

^c^ Intervention participants watched a video in addition to receiving a printed CRC screening brochure; Control participants only received printed materials

^d^ Intervention participants used a computer-based program that used MI and stages of change intervention related to CRC screening; Control participants used a computer-based program focused on diet and exercise

^e^ The nurses were provided with a number of tools to assist with offering the FOBT – including log sheets, visual aids, and video instructions

^f^ Interventions that were designed to leverage social network

**Table 4. Contextual Factors in Interventions to Improve FIT/FOBT screening for CRC, stratified by setting (Clinic-based, community-based, or both)**

| **Citation;**  **Study design** | **Clinic/Community history in improving CRC screening, length of partnership, predisposition to improving CRC screening** | **Clinic or Patient-level CRC Screening History at Baseline** | **Patient characteristics that modified intervention effect** | **Resources/Follow-up plan for positive stool screen** | **EMR Used** |
| --- | --- | --- | --- | --- | --- |
| ***Clinic-based Studies*** | | | | | |
| Baker, 2014 [40]  RCT | The OC-Light FIT became the new standard of care at the start of the study, replacing guaiac-based FOBT. | All pts had previously completed FOBT  The EFHC had initiated strategic CRC screening improvement prior to this study, which increased CRC screening from 17% in 2007 to 43% in 2009. This included (1) MAs identified patients due for screening, counsel them, and give them a home FOBT kit; (2) routine feedback to clinicians on their CRC screening rates; (3) CRC screening was added as a quality metric and (4) The EHR included clinical reminders for CRC screening. | The difference in benefit was largest among patients with no clinic visits during follow-up.  (P = .03 for interaction) | The navigator assisted with arranging diagnostic colonoscopy.  The EFHC provided free transportation for patients if necessary, and free colonoscopy  was available through the Northwestern Medical Faculty Foundation and Northwestern Memorial Hospital. | Yes, unspecified. |
| Coronado, 2011 [43]  RCT | A CDC-funded program was in place to provide follow-up colonoscopies for abnormal FOBT results at low cost to King County residents. | Prior screening NR. All patients were not up to date. | Reasons for reluctance:  1) FOBT was costly;  2) not enough time to do the test;  3) dietary and drug restrictions;  4) infrequent bowel movements;  5) competing health and personal issues. | Follow-up colonoscopies could be provided at low cost to patients with an abnormal FOBT result through a CDC funded pilot program serving King County residents and operated through the Breast and Cervical Health Program (BCHP). | Yes, unspecified. |
| Coronado, 2014 [61]  Non-randomized CT (pilot study) | Only 7.5% of participants (16/213) were found to have invalid address to the mailing. This may be due to a system-wide mailing to update patient address information that took place 3 months before the intervention. | The overall rate of fecal testing (gFOBT or FIT) at VGMHC was 5.1% in 2012. | The highest FIT rates occurred among patients aged 65 – 74; Hispanics; those whose primary language was Spanish;  and patients with 6+ clinic visits. | A local community organization in the Portland Metro area, Project Access Now, provided colonoscopies to uninsured patients with abnormal test results, through a coordinated network of volunteer providers. | Yes, unspecified. |
| Davis, 2013 [44]  RCT | NR | Baseline rates of CRC screening at 8 study clinics ranged from 1-2%. In study sample, 28% (262/961) of the patients had ever completed an FOBT | Patients with limited literacy were 1.6 (95% CI 1.169 to 2.16) times more likely to complete FOBT within 3 months in the nurse support arm compared with enhanced usual care (P = .002).  Patients with adequate literacy did not significantly differ in FOBT rates comparing the nurse support, education, and enhanced usual care arm. | If the results were positive, the nurse manager called patients to discuss the results, facilitate appointments with their primary care provider, and schedule patients for a diagnostic colonoscopy at the appropriate treatment center. | No, paper charts. |
| Friedman, 2001 [45]  RCT | NR | 18% (n = 27) of pts aged 55+ had sigmoidoscopy in the preceding 5 years. | NR | After the pt’s clinic visit, appts were made for returning the FOBTs to the clinic lab and for a follow-up visit to discuss the test results with the physician. | Presence or absence not specified. |
| Goldberg, 2004 [46]  RCT | The program has an electronic database of patients returning FOBT cards dating to the spring of 1999, the year prior to the study. | Prior screening NR. All patients were not up to date. | Patients with congestive heart failure (CHF) were less likely to return the cards:  0 of 7patients with CHF vs. 19 (41%) of 46 returned cards at the index appointment (*p=*0.04), vs. 22 (48%) of 46 over the study year (*p=*0.03). | Work-up is initiated for positive tests in collaboration with the primary care physician, as standard care. | Yes, unspecified. |
| Goldman, 2015 [47]  RCT | Same as [40] | None of the subjects had any prior CRC screening.  Same as [40].  Following system wide quality improvements in CRC screening, the proportion of patients up-to-date on CRC screening increased from 17 % in 2007 to 43 % in 2009. | FIT completion was more common for patients with more clinic visits (p<0.001).  Study authors note that  absolute rate of FIT completion and the marginal benefit of outreach were less than in their previous trial (37.3% vs. 82.2%), and postulate that the current sample is more resistant; the screening rate in the outreach group increased only slightly from 36.7 to 40.0% between months 6 and 12. | Plan not stated, although rates of follow-up colonoscopy are reported. | Yes, General Electric Centricity EHR. |
| Greiner, 2014 [48]  RCT | NR | Any prior screening NR; all patients were not up to date. | Factors with increased odds of completing CRC screening, AOR (95% CI):   - No prior DM dx: 1.76 (1.12, 2.76) - Prior asthma dx 2.71 (1.49, 4.91) - Never having heard that cutting on cancer causes it to spread: 1.74 (1.10, 2.75) - Self-efficacy: 1.56 (1.01, 2.43) - Specifying a test preference:   FIT: 1.91 (0.89, 4.10 )  CS: 4.07 (1.89, 8.80) | No plan specified. Costs for both FIT and colonoscopy covered to remove cost as a major barrier to screening. | Presence or absence not specified. |
| Gupta, 2013 [49]  RCT | To serve the uninsured, JPS offers a medical assistance program for uninsured residents of Tarrant County, including surgery and cancer care, on a sliding pay scale.  Usual care included office visit–based offers for screening with gFOBT, colonoscopy, sigmoidoscopy, or barium enema by primary care providers; no screening reminders were in use prior to the study. | Prior screening NR. All patients were not up to date. | Compared with colonoscopy, FIT outreach was associated with statistically significantly higher screening among men, women, whites, blacks, and Hispanics | Patients with an abnormal FIT result were referred for diagnostic colonoscopy, assisted with scheduling, provided bowel preps via mail or clinic pickup, appointment reminders, and preparation instructions. | Presence or absence not specified. |
| Hendren, 2014 [50]  RCT |  | Prior screening NR. All patients were not up to date.  Study authors note that very low baseline screening rate represents a primary care setting predominantly serving patients of low SES, at risk for omission of cancer screening. | Among patients who remained unscreened despite receiving the intervention, there was a non-significantly greater proportion of white patients, more low income patients, more uninsured patients and fewer Medicare patients. | NR | Yes, unspecified. |
| Jandorf, 2005 [51]  RCT | NR | Prior screening NR. All patients were not up to date. | NR | PN followed up FOBT results by confirming with participant that physician-referred FS had been scheduled and completed. | Presence or absence not specified. |
| Jean-Jacques, 2012 [52]  RCT | The clinic had an EHR system since 2006 and had been regularly tracking its CRC screening rate in accordance with the HRSA core measurement set guidelines. | Prior screening NR. All patients were not up to date.  The percentage of patients across all HIHC sites who were up to date with CRC screening was 17% in 2008, 36% in 2009. | NR | Patients were informed of their FOBT results by their primary care clinician by letter or phone according to usual clinic practice. Patients with positive FOBT results were referred for colonoscopy. | Yes, unspecified. |
| Lasser, 2011 [53]  RCT | NR | Prior screening NR. All patients were not up to date. | NR | Plan/resources not specified; reports diagnostic followup:  One control patient had a positive FOBT result that was not followed up with a colonoscopy; the remaining FOBT results were negative. | Yes, Epic Systems Corporation, Verona, Wisconsin. |
| Levy, 2012 [54]  Levy, 2013 [55]  RCT | NR | Based on self-report, the average rate of being up to date with screening across offices was 54.3% (standard deviation, 14.8%).  Colonoscopy was the most prevalent screening test among those excluded from the study (51.4% were up to date with colonoscopy; standard deviation, 14.8%), indicating that participating practices strongly preferred colonoscopy as a screening method. | Receiving doctor recommendation (OR, 1.7; 95% CI, 1.2–2.4), greater patient perceived importance of CRC screening (OR, 1.8 [scores of 8 –10 vs 1–7]); 95% CI, 1.2–2.5), and having a physical examination during the follow-up period (yes vs no; OR, 1.5; 95% CI, 1.03–2.1). | NR | Both, some clinics used paper charts and others used and EHR. |
| Potter, 2011 [56]  RCT | No prior CRC screening improvement efforts. | 21.4 vs 17.6% had FOBT in the past year: (p=0.077) | Men, Asian Americans, non-English speakers, and individuals with higher incomes were more likely than others to complete FOBT during the 6-month study period.  Participants with at least one emergency department visit in the past 2 years were less likely to complete CRC screening. | NR, but reports rates of colonoscopy between FLU-only and FLU-FOBT groups. | Both, NOS. Nurses determined dates of CRC screening tests by consulting either electronic or paper-based medical records. |
| Potter, 2011 [66]  Cohort study | No prior CRC screening improvement efforts. | 42.5 vs 43.9% had FOBT in the past year (P = ns) | Multivariate analysis for completing CRC screening,  OR (95% CI):  Primary language, Cantonese vs other: 1.92 (1.19–3.12) .  Had a mammogram or PSA in past 2 years:  1.60 (1.11–2.30) | No plan is mentioned, though abnormal FOBT results were reported to the clinic for follow-up by primary care clinicians. | Yes, unspecified. |
| Roetzheim, 2004 [57]  RCT | NR | At baseline, 22.1 (109/596) control vs 35.9% (180/600) intervention patients had FOBT in the past year (P < .0001) | Clinical predictors of FOBT screening,  OR (95% CI):  Age: 1.02 (1.002 to 1.04)  Charlson comorbidity: 0.86 (0.78 to 0.94)  Number of visits: 1.09 (1.05 to 1.12) | NR | Presence or absence not specified. |
| Singal 2015  RCT | NR | All patients were not up to date. | On multivariate analysis, screening participation was positively associated (OR (95% CI) with:  FIT: 3.84 (3.28-4.50)  CS: 1.83 (1.57-2.14)  Black race: 1.26 (1.06-1.49) Hispanic: 1.98 (1.71-2.29) Primary care contact:  1 visit: 2.92 (2.49-3.44)  2+ visits: 5.24 (4.55-6.05)  GI subspecialty care after randomization: 2.29 (1.40-  3.73)  “On interaction analysis, the effect of outreach did not appear to differ significantly by sex or race/ethnicity.” | NR | Presence or absence not specified. |
| Tu, 2006 [60]  RCT | No prior CRC screening improvement efforts, or special policies or procedures addressing CRC screening within the clinic. | Prior FOBT: 40% vs. 42% (P = .78) | No effect modification was found by age (P = .59), gender (P = .29), language (P =.82), insurance (P =.50), or prior FOBT (P =.99). | NR | Presence or absence not specified. |
| Tu, 2014 [64]  Pre-post, using cross-sectional data | No prior CRC screening improvement efforts within the clinic. | Up to date with FOBT screening at baseline: 148 (25%) vs. 69 (17%) | Patients who were nonadherent at baseline showed a significant increase in overall CRC screening (adjusted OR = 1.70; 95% CI 1.05, 2.75) at the intervention clinic compared to the control clinic. This difference was marginally significant (adjusted OR = 1.77; 95% CI 0.98, 3.18). | Colonoscopies were referred to specialists outside the community health center. | Yes, ICHS Electronic Medical Records. |
| ***Community-based Studies*** | | | | | |
| Braun, 2005 [41]  RCT | In the year prior to the study, project team conducted surveys and focus groups and with 56 Hawaii-based civic club members to examine CRC knowledge, attitudes, and behaviors. | Baseline screening rate,:  I vs. C: 59 vs. 69% were up-to-date with CRC screening.  30 vs. 39% had FOBT within past year. ^a^ | NR | Follow-up plan not described; reports that 9% of completed FOBTs were positive and were followed up. | No |
| Campbell, 2004 [42]  RCT | NR | 23.5 vs. 19.7 vs. 19.5 vs. 30.4% had FOBT in the past year (P = ns) | Limited, uneven distribution of LHA exposure through pre-existing social networks. Less than 10% of those in the LHA churches recalled talking with an LHA; those who did were more almost twice as likely to receive the intervention and complete FOBT. | NR | No |
| Larkey, 2006 [63]  Pre-post study | NR | 162 (87.1%) had never been screened.  24 (12.9%) had been screened but were out of compliance. | Among 24 women who had previously had FOBT and were out of compliance at baseline, only one (4.2%) obtained this test again during the time of the study; 3-month time frame might not have been long enough. | The authors established resources to follow up and arrange for endoscopy and/or treatment for positive FOBT tests. | No |
| Thompson, 2006 [59]  RCT | NR | Compliant with screening at baseline, by self-report:   - Hispanic: 61% - Non-Hispanic white: 48%   No prior history of screening:   - Hispanic: 49% - Non-Hispanic white: 39% | Among non-Hispanic Whites who had never been screened at baseline (N=604): 47.4 vs. 29.0% had used FOBT at final survey (P = .03) | NR | No |
| Wu, 2010 [65]  Pre-post study targeting patients | CRC screening was added to a successful 8-year program serving the state’s Asian American population through health fairs to recruit women for the Title XV breast and cervical cancer prevention program. (HAAP). As with other HAAP programs, the CRC education program had a coordinator from within the community. | 37% had ever been screened | The most common reasons for not having been screened were that they had no health insurance and could not afford it: “I don’t have a problem,” and “I only see a doctor when I am sick.” | Those with positive results were referred to their own physicians or a State of Michigan-provided list of free or low-cost community providers. | No |
| ***Combined Clinic- and Community-based Studies*** | | | | | |
| Redwood, 2011 [67]  Feasibility assessment | RFL was founded in 2004 by CRC survivor, Larry Holman. A core group of volunteers joined efforts to turn RFL into a cancer fundraiser.  RFL contacted the Anchorage Neighborhood Health Center (ANCH) to form a partnership to promote CRC screening among low-income and underin­sured ANHC patients. Previously there were no community-based CRC screening services available for low-income patients. ANCH was established in 1971 (it is an FQHC and one of Alaska’s largest primary care practices). | NR | NR | The ANHC screening care coordinator contacts patients with positive iFOBTs for follow-up.  Patients with insurance were referred to private endoscopy centers. Patients without insurance were eligible for RFL-funded colonoscopy; the ANHC screening care coordinator schedules colonoscopy with a participating gastroenterologist at a private endoscopy center, with preparation instructions. | No |
| Sarfaty, 2005 [62]  Sarfaty, 2006 [68]  Non randomized CT | The Maryland state cancer program was created in 2000, a year prior to the study. Colorectal cancer was chosen as a principal target. The state allocated funding to counties based on population and mortality rates, and required that each local health department establish a community coalition with broad participation in program planning. | Rates of prior FOBT by group:  Referred: 10%  Educator: 13%  Telephone: 14% | Factors most strongly correlated (*P* < .001) with screening completion by any modality:   - Usual source of medical care - Presence of symptoms | If FOBT results were positive, pts were contacted by the registered nurse to arrange a complete diagnostic examination by CS. | No |

**Table 5. Implementation strategies used in interventions to improve FIT/FOBT CRC screening, stratified by setting**

| **Citation; Study design** | **Inter-vention Source** | **Intervention Development** | **Intervention Training/Monitoring** | **Intervention Delivered By** | **Incentives at clinic and/or**  **Patient level** | **Cost** | |
| --- | --- | --- | --- | --- | --- | --- | --- |
| ***Clinic-based Studies*** | | | | | | | |
| Baker, 2014 [40] RCT | NR | The intervention was designed to address reasons for failure to adhere to annual FOBT. | NR | Project team searched the EFHC’s EHR data to identify eligible patients and to document their demographic characteristics, and randomly assign to intervention arms. All aspects of the intervention were implemented by clinic staff members (ie, health informaticist and care manager) using existing resources in the clinic (health information technology, automated calling and texting system). Outreach and specimen processing were performed centrally for all sites. [Clinic] | NR | The estimated cost of the outreach intervention was $34.59 per patient. Of the 202 patients who received outreach, 162 (80.2%) completed the FIT. Thus, the estimated cost per completed test was $43.13. | |
| Coronado, 2011 [43] RCT | Resear-cher | Working with the clinic, we designed a study in which we ascertained baseline rates of CRC screening (using clinic medical records only), conducted a clinic-based intervention, and examined post intervention rates of compliance with screening. | Training of the health promotors was conducted by the project PI who has much experience in this area. | One Spanish-speaking male health promoter was hired and trained to deliver the intervention. For the home visits, a Spanish speaking female medical assistant at Sea Mar accompanied the male health promoter to enhance acceptance of the household visits. The study team worked with the clinic to determine baseline rates of CRC screening using clinic medical records; conducted a post-intervention phone survey to assess intermediate outcomes related to CRC screening. [Clinic] | NR | NR | |
| Coronado, 2014 [61] Non-randomized CT (pilot study) | Resear-cher | Staff at participating clinics adapted existing workflows for use in the STOP CRC project. Study team convened a community advisory board; the board consisted of policy-makers, clinicians, patients and their advocates, and gastroenterologists. The board met 5 times throughout the year during a single 4-hour in-person meeting and 4–1.5 hour phone meetings. | The EHR Site Specialist who helped design the system trained clinic staff to use the EHR system. To minimize staff training at each site, patients were selected from a single provider team at each intervention site. Study team also held regular meetings of project investigators and clinic staff; bilingual project staff trained the Patient Care Coordinator in bilingual motivational interviewing techniques. | The Patient Care Coordinator at the Auto Plus clinic engaged patients using bilingual motivational interviewing techniques. [88] | NR | Actual costs NR. The clinic chose to pay for testing in uninsured patients, which meant that additional arrangements were made with the outside lab, so that patients with insurance could be billed directly and those without could be billed to the clinic. | |
| Davis, 2013 [44] RCT | Resear-cher | The educational strategy and nurse support arms were designed to overcome key patient barriers to CRC screening such as access to tests, limited knowledge, negative beliefs, and poor self-efficacy. Study team developed and piloted educational materials via focus groups and interviews with FQHC patients, providers, and community advisory boards. | Study personnel trained research assistants and nurse managers in a 2hr in-service session. RAs were trained in interviewing patients, administrating the survey and health literacy techniques. Nurse managers were trained in motivational interviewing, use of a tracking system, and patient navigation for positive results. | A medical assistant at each clinic identified potentially eligible patients. An RA then screened patients for eligibility and administered a structured interview. For the nurse support arm, the nurse manager engaged the patients using motivational interviewing techniques. [Both; mostly External] | Patients received $10 for participation in baseline survey. | The incremental cost of the educational intervention per additional individual screened was $250 over enhanced usual care, whereas the incremental cost of the nurse intervention per additional individual screened was $1337 over enhanced usual care. Incremental costs for the education arm were writing, producing, and editing a DVD ($10,000); brochures ($2000); and RA ($1036). Costs for the nurse arm were postcards ($60) and 40% of salaries for 2 nurses for 1 year ($106,280). | |
| Friedman, 2001 [45] RCT | Resear-cher | NR | NR | “…we recruited 160 patients from the waiting room….clinic physicians were aware that some of their patients might be participating in a study that would involve such a procedure.” A videographer worked with lay health educators and a physician to and produced the video used in the intervention [External] | NR | Not specified; mentions budgetary constraints as reason for the compromised sound quality of the video | |
| Goldberg, 2004 [46] RCT | Resear-cher | NR | NR | The colon cancer screening program is staffed by two nurse clinicians. Study investigators performed the data linkages that facilitate the mail-out and outcome assessment. Patients were approached in the waiting room and asked by the improvement project staff to complete a survey; this was used to create the project registry. Providers were unaware of the allocation of patients as study or control. [88] | NR | The costs of computer programing, staffing, and materials necessary to institute a system of mailing FOBT cards to the clinical population is estimated to be $1.00 or less for each set of cards mailed. Using estimates by Sonnenberg et al. for the number of years of life saved in a screening population, it would cost the current screening program approximately $3000 to $4000 more per year of life saved to systematically mail FOBT cards.” | |
| Goldman, 2015 [47] RCT | Resear-cher | “This study used similar methodology to a previous randomized controlled trial that sought to increase adherence to annual FOBT among individuals who were due for repeat CRC screening.” | NR | An EFHC programmer queried the EHR to identify eligible patients and collect analytic variables. Each week, patients randomized to the outreach group had FIT kits mailed to their homes, including provider letters and plain language FIT instructions. A few days after the mailing, an automated phone call, and 2 days later, a text message via a contracted commercial system. A commercial system was contracted for automated text messages. A CRC Screening Navigator is on staff at EFHC. A central EFHC laboratory processed all returned FITs and entered test results into the EHR. [Clinic] | NR | NR The authors note: “Future cost-benefit analyses are needed to determine the most successful and efficient strategies to initiate CRC screening for vulnerable populations.” | |
| Greiner, 2014 [48] RCT | Resear-cher | Formative research activities (interviews, usability testing) informed the development of all components and were used to target the touchscreen materials to the appropriate audience. | NR | Health center staff referred patients to the research staff at a kiosk in the waiting area. In the clinic waiting area, research staff administered a verbal screening survey to determine patients’ study eligibility, administered the intervention in clinic waiting room, and provided FIT kits or scheduled colonoscopies after the health provider visit. [External] | Clinic-level: NR.  Patients: Reimbursed with a $20 gift card and mailed an additional $20 gift card after completion of the follow-up survey. | Amount not stated. Costs for both FIT and colonoscopy were covered by the study in an effort to remove cost as a major barrier to obtaining screening. | |
| Gupta, 2013 [49] RCT | Resear-cher | NR | NR | “Delivering organized screening required nearly full-time efforts of a medical assistant and a nurse. Whether these efforts can be resourced and implemented sustainably requires further study.” [Unclear, but likely clinic staff rather than research staff] | NR | NR | |
| Hendren, 2014 [50] RCT | Resear-cher | NR | NR | Written prompt sheets for providers and patients were developed and delivered to the practice each week for MAs to distribute at the time of a patient appointment, if randomized to the Tx group. Reminder materials distributed by MA/clinicians to patients (but prepared in advance by study team). An outreach worker (not clinic staff) mailed a personalized letter to each intervention patient. Written prompt sheets reminded both the clinician and patient that the patient was past due for CRC screening. Reminder calls made by an automated system.  [88] | NR | The cost per letter mailed was approximately $1.90, including material and labor costs. The total cost for the automated calls was about $0.92, including the preparation of each list of call recipients from the database and the monitoring of post-call status. The FIT kits cost about $25.12, including the Medicare reimbursement rate for the kit and the preparation for mailing/mailing costs. Finally, a small labor cost was incurred for using the point-of-care prompts. Medical assistant wages are about $14.48 per hour (http://www.bls.gov/oes/current/oes319092.htm). Using the prompts is estimated to take about 15 min per day of a medical assistant’s time, so for a practice about $3.62 per day in staff time would be needed to distribute the written prompts. | |
| Jandorf, 2005 [51] RCT | Resear-cher | The East Harlem Partnership for Cancer Awareness was established to help identify barrier to cancer screening in minority, medically underserved people in the East Harlem community. Information obtained during 5 focus group sessions suggested that one possible barrier to CRC screening in this population is the difficulty patients have trying to navigate the health system. Therefore, we reasoned that a patient navigator system might enhance the completion of CRC screening tests. | NR | The RA served as the Patient Navigator. The RA approached prospective participants in English or Spanish, as preferred. [External] | NR | NR | |
| Jean-Jacques, 2012 [52] RCT | Resear-cher | NR | A script was developed to guide the outreach calls. One of the project authors trained and supervised the outreach coordinator. | All print materials were provided in English and Spanish, and the outreach coordinator used a telephone-based interpretation service to communicate with patients who were not proficient in English or Spanish. Does not specify who performed the FOBT mailings, but presumably done by project staff. FOBT cards were processed by the health center laboratory staff according to usual protocol. [88] | NR | NR. Study did not include formal cost or cost-effectiveness analyses. | |
| Lasser, 2011 [53] RCT | Resear-cher | Several nonrandomized studies, including our own, have shown that patient navigation can increase rates of CRC screening among urban, racial minority patients….to build on this limited research we conducted a RCT of patient navigation that included immigrants at various safety net clinics. The intervention was piloted in 1 health center. | The navigators attended a 2-day training that included role-play scenarios on motivational interviewing; CRC prevention, logistics of FOBT, colonoscopy. The project manager audited at least 5 patient calls by each navigator for adherence to a calling script and for motivational interviewing techniques. The patient navigators and the project manager also met on a weekly basis to discuss challenges arising during the outreach calls and to review the use of motivational interviewing techniques | Internal staff included 3 trained navigators who were fluent in English and Spanish, Portuguese, or Haitian Creole were based centrally in the Departments of Medicine and Community Affairs, one was a CNA and the other two were experienced community health workers. Research team - project manager [External] | NR | NR | |
| Levy, 2012 [54] Levy, 2013 [55] RCT | Resear-cher | NR | NR | Practices provided a list of patients in target age range. Research team handled all study elements except placement of reminder in clinics with a paper chart which was performed by the office manager. The physician reminder was placed in charts of pts who agreed to be in the study. The reminder was programmed if EHR was used. [Externally coordinated; but involved clinic in placing physician chart reminder] | Each clinic received $1000 per year for providing patient lists for up to 3 years.  Local clinic coordinators received $200 for completing certification for working with human subjects.  Subjects received $20 for completion of the baseline questionnaire and $10 for completion of the educational assessment questionnaire. | NR | |
| Potter, 2011 [56] RCT | Resear-cher | One health department clinic had participated in a pilot study of the intervention; other sites invited to participate (see Potter 2011, #794) | Prior to the study, the research team provided a 1-hour training session for nursing staff. The week before the beginning of FLU season, one research assistant visited each site to observe baseline clinic processes and patient flow and to review study procedures individually with each nurse. During the study, three RAs made frequent visits to each of the six study sites to ensure that clinic nurses were aware of that week’s study procedures and to make observations of the implementation process. | The medical director of each clinic provided standing orders to nurses (including licensed vocational nurses and MAs) to provide FLU to age eligible primary care patients. However, nursing staff could decline to offer FOBT if they judged a patient to be too ill to accept, if the clinic was short staffed, or if they were too busy with other orders to allow time for the intervention. Nurses were given tools to assist, including FLU-FOBT log sheets, visual aids, written/video instructions, stamped envelopes for returning completed FOBT to lab. RAs were available on a daily basis to answer nurses’ questions regarding study implementation. [Clinic] | Clinic promised $5000 honorarium to be given at end of study regardless of results achieved. | NR | |
| Potter, 2011 [66] Cohort study | Resear-cher | The researchers met with the medical leaders of CPHC to discuss FLU-FOBT Program procedures. Key FLUFOBT Program elements requested by the CPHC leadership program reminders including flu shot log and FOBT information sheets targeting individuals with low literacy, printed in Chinese and English. Nursing stations were equipped with a four-minute video in Cantonese on FOBT that could be played while nurses prepared the flu shots. | Nursing staff attended a one-hour group training session. A bicultural bilingual Cantonese co-investigator provided on-the-job training during the first few days of the study and made visits to record observations for evaluation purposes. | Nursing staff determined eligibility using electronic clinical data system. Nursing staff were encouraged to follow the FLU-FOBT protocol whenever possible, but were also given the freedom to decide how to discuss FOBT with patients and which educational approaches or materials to use. They were allowed to bypass offering flu shots or FOBT when they were too busy. [Clinic] | Clinic promised $5000 honorarium to be given at end of study regardless of results achieved. | NR | |
| Roetzheim, 2004 [57] RCT | Resear-cher | NR | Research team trained office staff and providers in a 45-min session; conducted unannounced audits of a random sample of 25 charts of eligible patients seen in the most recent week; compliance with the system was assessed during formal feedback sessions with clinic staff every 6 months; and assessed control clinics for possible contamination. | Receptionists provided eligible pts with the cancer-screening checklists. Nursing staff reviewed the completed checklist and attached it to pt chart. Providers reviewed the checklist and used colored stickers to indicate cancer screening action. Both nurses and providers were responsible for updating the appropriate colored chart stickers. During feedback sessions office staff and project staff jointly discussed how the intervention was proceeding, what problems were occurring, and what might be done to improve implementation. Research assistants abstracted patient records to determine intervention effectiveness. [Clinic] | NR | Total cost per patient: $ 2.96 for FOBT intervention. Incremental effectiveness of $11 for CRC screening. | |
| Singal, 2016 RCT | Resear-cher | NR | Research staff were trained to conduct telephone calls in English or Spanish to invite participation using standardized scripts; also trained to call patients before CS to give preparation instructions. | External research staff. [External] | NR | Patients had a $25 copay for CS. Costs not otherwise specified. | |
| Tu, 2006 [60] RCT | Resear-cher | This study was conducted in collaboration with ICHS...We developed educational materials based on findings from our earlier qualitative interviews and focus groups. | NR | “We” used ICHS administrative computer database to identify age-eligible patients, who were sent a bilingual letter, in Chinese and English, from the medical director to introduce the study. During patient visits, a bicultural health educator (who had worked at the Clinic for several years as a MA) reviewed records to confirm patient eligibility. The health educator approached patients during visit for study participation; and followed a randomization table to assign patients into intervention or control in chronological order. [Both; mostly clinic] | NR | NR | |
| Tu, 2014 [64] Pre-post, using cross-sectional data | Resear-cher | This research was conducted in collaboration with ICHS…we adapted our original evidence based intervention with: MAs serving as the intervention agents instead of a health educator; no FOBT kits provided by the MAs as consistent with ICHS procedures; and a series of brief in-service presentations to the MAs. | Project staff delivered 15 in-service presentations to the MAs, and 2 to all staff at the intervention clinic. | MAs were asked to distribute the intervention materials (translated into Vietnamese) to Vietnamese patients who appeared to be age eligible. MAs were specifically informed that they were not expected to provide health education. [88] | NR | NR Authors state: “...adapting our intervention agent from a health educator to MAs provided economic advantages as well as better compatibility, simplicity, and trialability. Using less specialized MAs represents an economic advantage over using health educators.” | |
| ***Community-based Studies*** | | | | | | | |
| Braun, 2005 [41]  RCT | Resear-cher | Study team partnered with Association of Hawaiian Civic Clubs. Developed intervention based on preliminary study using focus groups and survey methodology, results suggested that a culturally targeted intervention for Native Hawaiians could be strengthened by incorporating concepts from social learning theory (SLT). | NR | Control arm: A non-Hawaiian nurse delivered a targeted educational presentation. Intervention arm: A Native Hawaiian physician delivered the targeted educational presentation. A Native Hawaiian CRC survivor also told his personal story. Native Hawaiians chosen to deliver these messages were individuals about whom civic club members voiced strong approval and respect.  [Both] | NR | | NR |
| Campbell, 2004 [42]  RCT | Resear-cher | Early in the project focus groups were conducted in two pilot churches to identify concerns, barriers, beliefs, and motivators for desired behaviors. Findings revealed that participants lacked knowledge about the gastrointestinal tract and CRC and that their health providers had not recommended CRC screening. Findings informed the study intervention and measures. | Project staff conducted the LHA training sessions. | Recruited culturally specific facilitators/ lay health advisors (LHAs) from within the church community.  A professional videographer produced the videotape which was targeted to an African American church audience. Community members and pastors provided testimonials in the newsletters, and were featured in the TPV videotapes.  [Community] | Churches were given a donation from the project of $300 on enrollment in the study and a second donation of $300 after the completion of the follow-up surveys. | | The mailed, home-based intervention cost approximately $20 per person, not including development costs.  Other costs NR. |
| Larkey, 2006 [63]  Pre-post study | Resear-cher | Three Latina community health advisors, or Promotoras de Salud, assisted in developing curricula for five cancer prevention and screening objectives. | NR | Promotoras utilized networks of contacts in churches and community centers to promote the program and recruit women from underserved areas.  [Community] | NR | | NR |
| Thompson, 2006 [59]  RCT | Resear-cher | Study team conducted focus groups to  develop and test our intervention activities. The study team recruited a community advisory board (CAB) who provided insights to the cultural appropriateness of different intervention activities. | Research team trained project staff on approaching households, asking questions in a standard manner, editing questionnaires, and documenting household contacts and surveys. | Research team hired local bilingual interviewers to conduct in-person surveys. Project staff recruited a Community Advisory Board; organized community-level activities and health promotion events.  [Community] | Clinic: NR  Respondents were given a $5 grocery store coupon. | | NR |
| Wu, 2010 [65]  Pre-post study targeting patients | Resear-cher | CRC screening was added to a successful 8-year program serving the state’s Asian American population through health fairs to recruit women for the Title XV breast and cervical cancer prevention program. (HAAP). | NR | As with other HAAP programs, the CRC education program had a coordinator from within the community. Expert presenter for educational seminar; lay health advisor for recruiting study participants thru health fairs; volunteer medical and nursing students from within Asian American communities did screenings at health fairs.  The coordinator conducted outreach via community gathering places (e.g., churches, temples, community centers, group picnics).  [88] | NR | | NR |
| ***Combined Clinic- and Community-based studies*** | | | | | | | |
| Redwood, 2011 [67]  Feasibility assessment | Com-munity | RFL was founded in 2004 by CRC survivor. A core group of volunteers developed RFL further as a cancer fundraiser. | NR | RFL formed a partnership with ANHC to promote CRC screening among low-income and underin­sured.  Event coordination included gathering permits and insurance, advertising with bicycle clubs and outdoor groups, and setting up camping space, food, rest stops, support stations, and transportation for partici­pants back to Anchorage after the ride.  RFL and ANHC worked with gastroenterologists, medical practices, and pathology services to contribute pro bono and reduced-fee services for CRC screening. RFL funds were used for a part-time staff position at ANHC for a screening care coordinator  [Community] | Patients who bring back their completed iFOBT receive a $5 grocery store gift card. | | The program began outreach efforts to patients in December 2007 with $35,000 in funding from RFL; in 2008, RFL contributed $60,000. |
| Sarfaty, 2005 [62]  Sarfaty, 2006 [68]  Non randomized CT | County govern-ment | Montgomery County established a colorectal cancer education, screening, and treatment program as part of the state cancer program. The program provided free colonoscopy to uninsured county residents. | NR | County program staff; the community campaign included private and public partners including 4 hospital systems, 3 minority health groups, a coalition of community clinics, a hospice association, the local chapter of the ACS, and a Housing Opportunity Commission residence. Several partners hired certified health educators. The state and county sponsored training workshops. The Latino Health Initiative hired and trained promotoras. The clinical program was contracted to a nonprofit partner, which subcontracted with physicians, hospitals, and laboratories and reimbursed services.  [Both; County and community] | Clinic: NR  Patient: NR  Coalition partners received funding to participate. | | The education and outreach program educated 11,886 people at a cost of $122 per individual educated.  The clinical screening program provided screenings to 866 at a cost of $1688 per individual. |
